# Campania Preventability Assessment Committee (Italy): A Focus on the Preventability of Non-steroidal Anti-inflammatory Drugs' Adverse Drug Reactions

**DOI:** 10.3389/fphar.2017.00305

**Published:** 2017-05-26

**Authors:** Maurizio Sessa, Liberata Sportiello, Annamaria Mascolo, Cristina Scavone, Silvia Gallipoli, Gabriella di Mauro, Daniela Cimmaruta, Concetta Rafaniello, Annalisa Capuano

**Affiliations:** Section of Pharmacology “L. Donatelli”, Department of Experimental Medicine, Campania Pharmacovigilance and Pharmacoepidemiology Regional Centre, University of Campania “Luigi Vanvitelli”Naples, Italy

**Keywords:** preventability, spontaneous reporting system, pharmacovigilance, medication errors, Italy, humans, drug safety, adverse event

## Abstract

**Purpose:** This study aims to investigate preventability criteria of adverse drug reactions (ADRs) involving non-steroidal anti-inflammatory drugs (NSAIDs) by analyzing individual case safety reports (ICSRs) sent through Campania region (Italy) spontaneous reporting system from July 2012 to October 2016.

**Methods:** For all the ICSRs that reported NSAIDs as suspected drug, a trained multidisciplinary team of Campania Pharmacovigilance Regional Centre composed of clinical pharmacologists and pharmacists with pluriannual experience in Pharmacovigilance assessed preventability by using the P-method.

**Results:** In all 19,039 ICSRs were sent to Campania Pharmacovigilance Regional Centre, of which 550 reported NSAIDs as suspected drug. In total, 94 cases (17.1%) out of 550 ICSRs were preventable. In the 94 preventable cases, 201 critical criteria were detected of which 182/201 (90.5%) related to healthcare professionals' practices, 0/201 (0.0%) to drug quality, and 19/201 (9.5%) to patient behavior. The most detected critical criteria were the necessary medication not given (52/182; 28.6%), labeled drug–drug interaction (36/182; 19.7%), incorrect drug administration duration (31/182; 16.9%), wrong indication (26/182; 14.2%), therapeutic duplication (18/182; 10.0%), and documented hypersensitivity to administered drug or drug class (10/182; 5.6%). In seventeen (18.1%) preventable cases, there were 19 critical criteria involving non-compliance (15/19 critical criteria; 78.9%) and self-medication with the non-over-the-counter drugs (4/19 critical criteria; 21.1%). In all, 17 out 94 (18.1%) preventable cases involved over-the-counter drugs.

**Conclusion:** A call for action for Campania Pharmacovigilance Regional Centre is necessary in order to promote initiatives to increase the awareness of healthcare professionals and citizens on the risk associated with inappropriate use of NSAIDs.

## Introduction

Due to their ability to relieve pain and reduce inflammation, non-steroidal anti-inflammatory drugs (NSAIDs) remains among drug class mostly used in the world (Laine, 2001). Thus, not surprisingly, NSAIDs are listed among drugs with a high prevalence of inappropriate drug use, a type of medication error (Kovac et al., 2010; Ussai et al., 2015), which lead to preventable adverse drug reactions (ADRs) (Kunac and Tatley, 2011). As the result of initiatives to improve the identification of medication errors in the individual case safety reports (ICSRs) promoted by World Health Organization (Pal et al., 2015), and recently strengthen in Europe (European Medicine Agency, 2011, 2013), committees were introduced in Italy to meet these new regulatory tasks (Bencheikh and Benabdallah, 2009; Sessa et al., 2016a,d). Although on the Italian national territory evidence was provided regarding the association between inappropriate use of NSAIDs and the risk of experiencing serious and non-serious ADRs (Montagnani et al., 2016; Rafaniello et al., 2016), no studies were conducted to assess the magnitude of their preventability, highlighting the need of gain further insight on this aspect. This is even more important considering that on the National territory, several NSAIDs were sold as over-the-counter medications, which dispensing profile is well known to lead to abuse in their usage, with harms being increasingly recognized (Cooper, 2013). Given that little is known regarding the preventability of ADRs occurred during NSAIDs use, as well as the impact of dispensing procedure on this phenomenon, this study aimed to investigate preventability criteria of ADRs involving NSAIDs as suspected drugs in ICSRs sent through Campania region spontaneous reporting system.

## Methods

### Data sources

For the purpose of this study, all ICSRs sent through Campania Region spontaneous reporting system and collected in the Italian Pharmacovigilance Network database from July 2012 to October 2016 were screened to select those that reported as suspected drug NSAIDs (Anatomical Therapeutic Chemical classification, ATC code M01, excluding M01AH).

### Preventability assessment

In our study, for all the ICSRs that reported NSAIDs as suspected, a trained multidisciplinary team of Campania Pharmacovigilance Regional Centre composed of clinical pharmacologists and pharmacists with pluriannual experience in Pharmacovigilance assessed preventability by the P-method as described elsewhere (Benkirane et al., 2015; Sessa et al., 2016a,d). The “P-method,” is an algorithm that was developed to assess the preventability of ICSRs sent through spontaneous reporting system and that was validated by several Pharmacovigilance centers in the program for International Drug Monitoring coordinated by the World Health Organization (Benkirane et al., 2015; Pal et al., 2015).

The evaluation of preventability is a biomedical methodology that consists of three steps. The first step is the causality assessment evaluation that in our study was performed by using the Naranjo algorithm (Naranjo et al., 1981) as in accordance with the standard national procedure suggested by Italian Medicine Agency (AIFA). The second and third steps are the determination of the potential mechanism for the ADRs, and screening of the critical criteria listed in the P-Methods (Benkirane et al., 2015), respectively. Evaluation of the critical criteria consists in answering a questionnaire (Supplementary Table 1), at which the multidisciplinary team could answer positively, negatively or that the question was “not applicable” or “unknown.” A positive answer (each question represent a critical criterion) made the case eligible to be classified as preventable. More than one critical criterion is potentially detectable. No positive answer for any of the critical criteria made the case eligible to be classified as not preventable. Cases with insufficient information to assess critical criteria were classified as not assessable. Only for those cases with a causality assessment at least “Possible” on Naranjo algorithm scale, critical criteria for preventability were evaluated. For each case and for each phase of the preventability assessment, whenever a full agreement was not reached, the multidisciplinary team based the final decision on a majoritarian system. Summary of Product Characteristics (SmPCs) published by AIFA were used for all evaluations that required information provided in SmPCs. ICSRs were classified as “documented” and “non-documented” based on the procedure described by Benkirane et al. (2015).

### Descriptive analysis and case series

Clinical and demographic characteristics of cases undergone to preventability assessment, including the type of reporter, seriousness, and outcome of ADRs were provided for descriptive purposes both for “preventable” and “not preventable” cases. Only for preventable cases instead, a case series and reporting rate were provided. Reporting rate was assessed by dividing the number of preventable ICSRs for the age/gender-weighted Campania Region's NSAIDs drug daily dose (DDD) for 1,000 citizens per die (DDD/1000 ab die). For this purpose, the Campania Region's NSAIDs DDD/1,000 ab die which were estimated by AIFA and which were reported in the “National report on Medicines use in Italy” published in 2015 were used (The Medicines Utilisation Monitoring Centre., 2015). For 2016, instead, no estimation of Campania Region's NSAIDs DDD/1,000 ab die was available. Therefore, it was not possible to assess the reporting rate. The role of DDD/1,000 ab die as drug utilization measure is described elsewhere (Wessling and Boethius, 1990). Seriousness was codified in agreement with the International Council on Harmonization E2D guidelines^1^. For outcome instead, six categories were used: recovered, improvement, resolution with sequelae, unchanged clinical condition, death, and not available.

### Sensitivity analysis—NSAIDs that did not require medical prescription during the study period

A sensitivity analysis was performed to provide an overview of preventability criteria and clinical scenarios found among preventable cases that involved NSAIDs that did not require medical prescription during the study period. For this purpose, for all preventable cases, an analysis of dispensing procedure for NSAIDs reported in the ICSRs was performed by using an open access database (https://www.federfarma.it/Farmaci-e-farmacie/Cerca-un-farmaco.aspx) provided by Federfarma. Federfarma is the national federation of pharmacy owners functioning as the representative of the sector toward National institutions.

## Results

In the period from July 2012 to October 2016, 19,039 ICSRs were sent to Campania Pharmacovigilance Regional Centre, of which 550 reported NSAIDs as suspected drug. Hospital physicians (gastroenterologist) were the main reporters, sending 344 (62.5%) out of 550 ICSRs. Causality assessment resulted probable for 325 (59.1%) cases and possible for 225 (40.9%) cases, therefore, preventability assessment was performed for all cases. All cases had both narrative and documental information required for the case evaluation.

### Preventable cases

**Figure 1:**
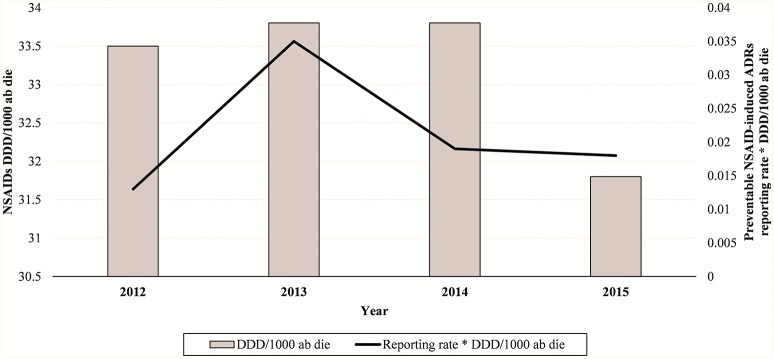
**NSAIDs-induced preventable ADRs reporting rate in Campania Region (Italy) from 2012 to 2015**.

**Figure 2:**
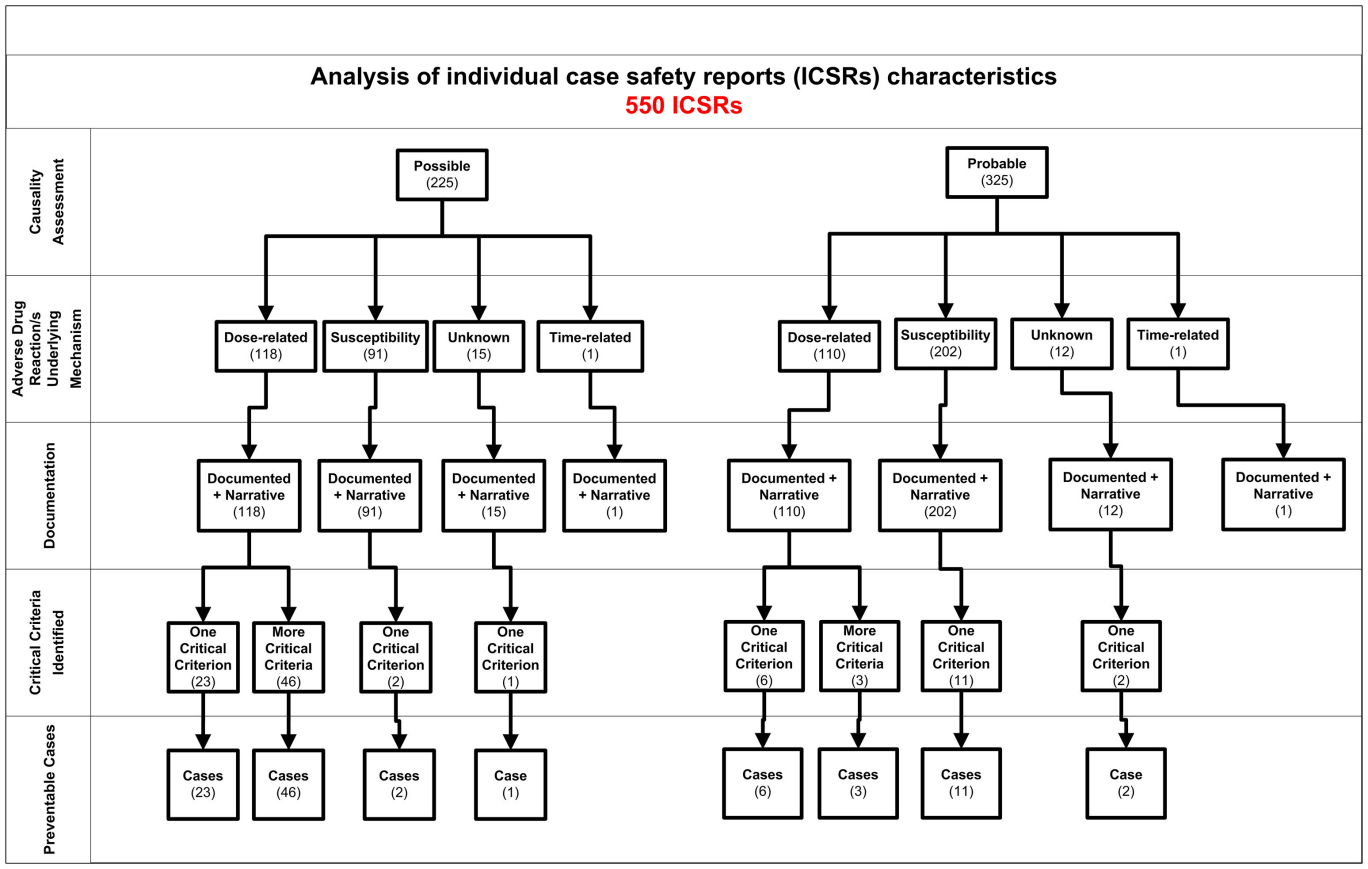
**Characteristics of individual case safety report involving NSAIDs recognized in Campania spontaneous reporting system from July 2012 – October 2016**.

In total, 94 cases (17.1%) out of 550 were preventable according to at least one critical criteria of the P-method, while 456 reports (82.9%) were not preventable. All the members of the multidisciplinary team for preventability assessment agreed for all preventable cases. Characteristics of preventable and not-preventable cases were presented in Table 1. Preventable cases reporting rate ranged from 0.013 ICSRs per DDD/1000 ab die in 2012 to 0.035 ICSRs per DDD/1000 ab die in 2013 (Figure 1). In 78 (83.0%) out of 94 preventable cases, the underlying mechanism of ADRs was dose-related, while in sixteen preventable cases the underlying mechanism of ADRs was susceptibility (13; 13.8%) and unknown (3; 3.2%), respectively (Figure 2). In the 94 preventable cases, 201 critical criteria were detected of which 182/201 (90.5%) related to healthcare professionals' practices, 0/201 (0.0%) to drug quality, and 19/201 (9.5%) to patient behavior. In 80 (85.1%) out of 94 cases, pharmacological and/or non-pharmacological treatments, drug switch or withdrawal were required. In all 52 out of 94 (55.3%) preventable cases required a hospitalization.

**Table 1:** **Demographic characteristics and distribution for the type of reporter, documentation, seriousness, outcome, causality assessment, and miscellaneous information of preventable and not preventable cases involved NSAIDs drugs recognized in Campania spontaneous reporting system from July 2012 – October 2016**.

**Variable**	**Level**	**Not preventable (*n* = 456)**	**Preventable (*n* = 94)**	**Total (*n* = 5 50)**
Gender	Female	258 (56.6)	45 (47.8)	303 (55.1)
	Male	188 (41.2)	48 (51.1)	236 (42.9)
	Not available	10 (2.2)	1 (1.1)	11 (2.0)
Age	Mean (standard deviation)	43.2 (19.8)	52.7 (17.5)	44.8 (19.7)
Number of reported drugs	>1	128 (28.1)	73 (77.7)	201 (36.5)
	1	328 (71.9)	21 (22.3)	349 (63.5)
Seriousness	Serious–other clinically significant condition	58 (12.7)	15 (16.0)	73 (13.3)
	Serious–death	1 (0.2)	0 (0.0)	1 (0.2)
	Serious–hospitalization	64 (14.0)	52 (55.3)	116 (21.1)
	Serious–life threatening	10 (2.2)	4 (4.3)	14 (2.5)
	Not defined	4 (0.9)	2 (2.1)	6 (1.1)
	Not serious	319 (70.0)	21 (22.3)	340 (61.8)
Outcome	Death	1 (0.2)	0 (0.0)	1 (0.2)
	Improvement	244 (53.5)	60 (63.8)	304 (55.3)
	Unchanged clinical condition	5 (1.1)	1 (1.1)	6 (1.1)
	Not available	34 (7.5)	16 (17.0)	50 (9.1)
	Recovered	156 (34.2)	13 (13.8)	169 (30.7)
	Resolution with sequelae	16 (3.5)	4 (4.3)	20 (3.6)
Reporter	Other healthcare professions	0 (0.0)	1 (1.1)	1 (0.2)
	Pharmaceutical industry	3 (0.7)	0 (0.0)	3 (0.5)
	Anti-poison center	1 (0.2)	0 (0.0)	1 (0.2)
	Pharmacist	118 (25.8)	16 (17.0)	134 (24.4)
	Nurse	19 (4.2)	1 (1.1)	20 (3.6)
	General practitioner	4 (0.9)	2 (2.1)	6 (1.1)
	Hospital physician	275 (60.2)	69 (73.3)	344 (62.5)
	Nor defined	0 (0.0)	1 (1.1)	1 (0.2)
	Patient	29 (6.4)	1 (1.1)	30 (5.5)
	Pediatrician	3 (0.7)	0 (0.0)	3 (0.5)
	Specialist	4 (0.9)	3 (3.2)	7 (1.3)
Causality	Possible	153 (33.6)	72 (76.6)	225 (40.9)
	Probable	303 (66.4)	22 (23.4)	325 (59.1)
Documented	Yes	456 (100.0)	94 (100.0)	550 (100.0)
Action taken	Yes	393 (86.2)	80 (85.1)	473 (86.0)
	No	63 (13.8)	14 (14.9)	77 (14.0)

### Preventable cases with critical criteria related to healthcare professionals' practices

**Figure 3:**
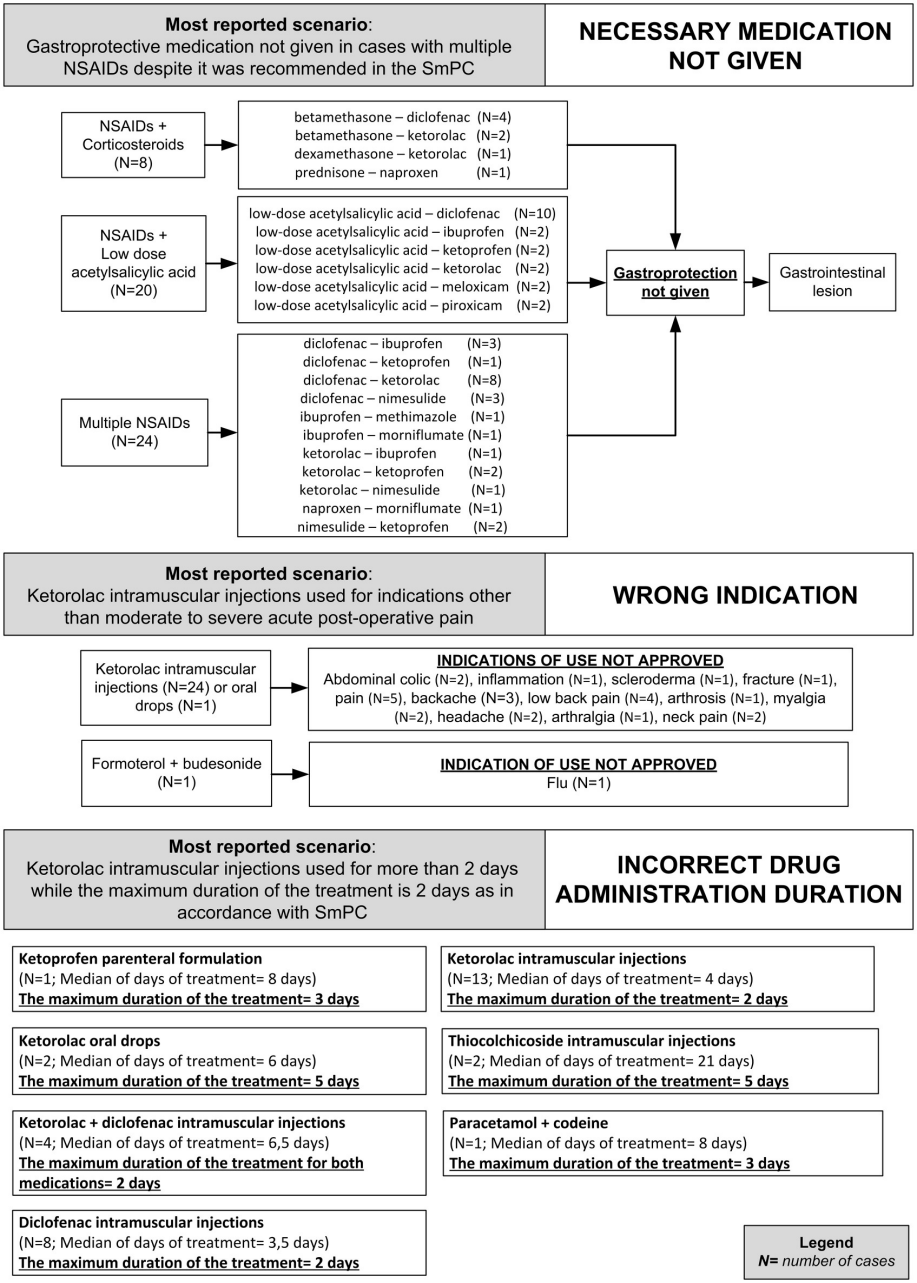
**Clinical scenarios of preventable cases reporting NSAIDs as suspected drug recognized in Campania spontaneous reporting system from July 2012 to October 2016 in which were detected critical criteria involving healthcare professionals' practices - part I**.

**Figure 4:**
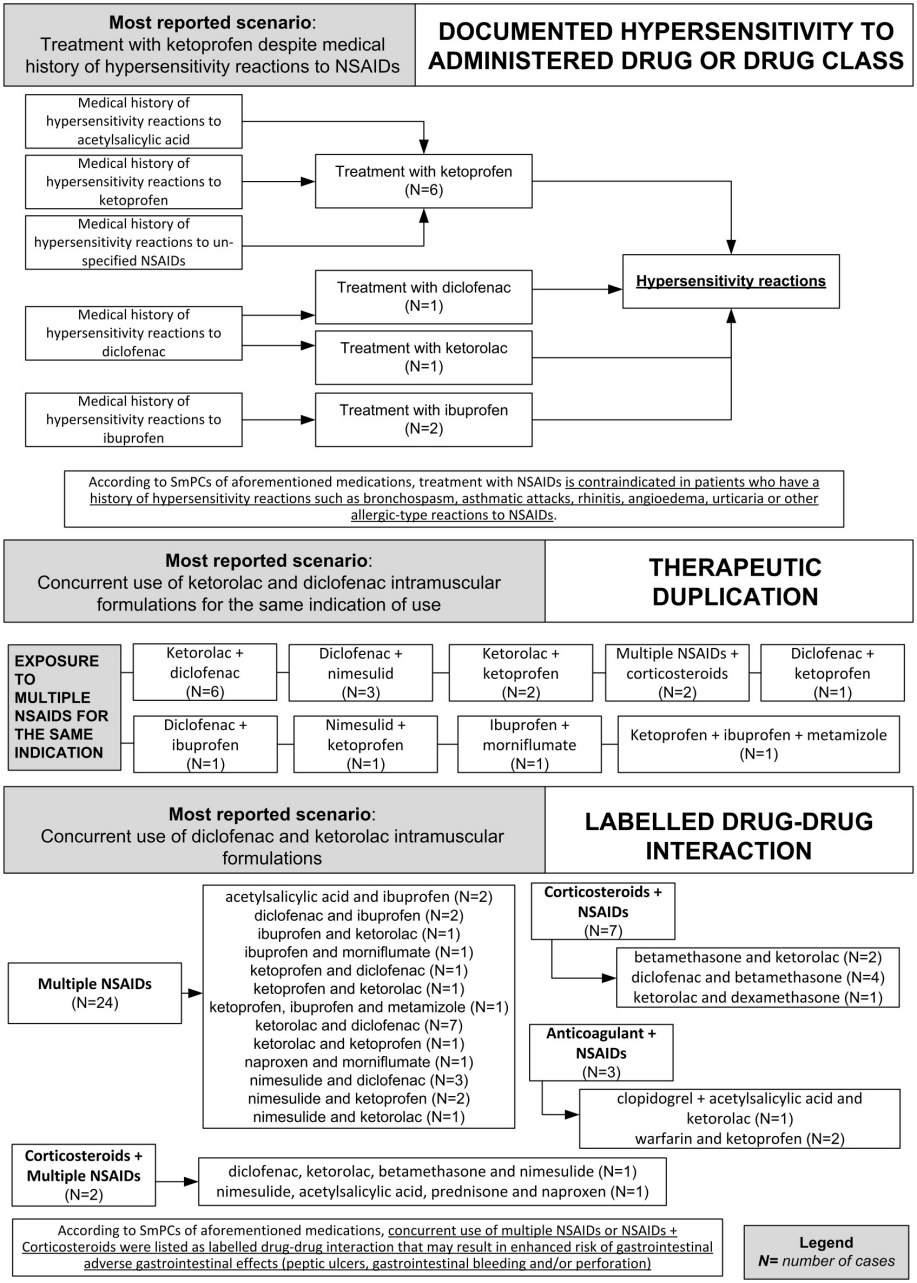
**Clinical scenarios of preventable cases reporting NSAIDs as suspected drug recognized in Campania spontaneous reporting system from July 2012 to October 2016 in which were detected critical criteria involving healthcare professionals' practices - part II**.

In 81 (86.2%) preventable cases, 182 critical criteria related to healthcare professionals' practices were identified. The most detected critical criteria were the necessary medication not given (52/182; 28.6%), labeled drug–drug interaction (36/182; 19.7%), incorrect drug administration duration (31/182; 16.9%), wrong indication (26/182; 14.2%), therapeutic duplication (prescription of two or more medicines with similar ingredients; 18/182; 10.0%), and documented hypersensitivity to administered drug or drug class (10/182; 5.6%). The most reported clinical scenarios involving aforementioned critical criteria are presented in Figures 3, 4.

Critical criteria related to healthcare professionals' practices included six (3.3%) cases for which were detected “incorrect dose” as a critical criterion and three (1.7%) cases with “inappropriate prescription for patient's underlying medical condition (renal failure, hepatic failure, etc.) underlying pathology” as a critical criterion. The clinical scenarios involving “incorrect dose” as critical criterion included one case using a non-therapeutic dosage of ketoprofen (340 mg/os/die for 2 days), while in adults, according to the SmPC, the maximum suggested dosage is 240 mg/os/die. Other two cases included the use of nimesulide 400 mg per os and nimesulide 300 mg per os, respectively, while according to the SmPC, the maximum suggested dosage of nimesulide is 200 mg/os/die. Moreover, a case using 175 mg of the combination clopidogrel + acetylsalicylic acid, as opposed to the SmPC for which the maximum suggested dosage of clopidogrel + acetylsalicylic acid in adults is 75 mg. Finally, a case using diclofenac 200 mg/Kg for 2 days, while according to the SmPC, the maximum suggested dosage of diclofenac is 200 mg/os/die.

The clinical scenario involving “inappropriate prescription for patient's underlying medical condition (renal failure, hepatic failure, etc.) or underlying pathology” as critical criterion included a case of a patient affected by liver cirrhosis treated with a parenteral formulation of ketorolac for arthrosis, while according to ketorolac SmPC severe liver disorder represent a contraindication for ketorolac administration. Moreover, a case of a patient affected by erosive gastroduodenitis treated with intramuscular injections of diclofenac 75 mg while according to diclofenac SmPC, erosive gastroduodenitis represents a contraindication for the administration of diclofenac. Finally, a case of a patient affected by liver cirrhosis that was treated with diclofenac 150 mg per os, while according to diclofenac SmPC, severe liver disorders are a contraindication for the administration of diclofenac. All preventable cases with healthcare professionals' practices as critical criteria are shown in Supplementary Table 2.

### Preventable cases with critical criteria related to patient behavior

In 17 (18.1%) preventable cases, there was, at least, one critical criterion related to patient behavior with 19 critical criteria involving non-compliance (15/19 critical criteria; 78.9%) and self-medication with non-over-the-counter drug (4/19 critical criteria; 21.1%).

The clinical scenarios involving critical criteria related to patient behavior included the concurrent use of over-the-counter medical products containing ibuprofen (5/19 critical criteria) or ketoprofen (1/19 critical criteria) concurrently with low-dose acetylsalicylic acid. According to SmPCs of both medications, concurrent use of an NSAID with low-dose acetylsalicylic acid made the cases eligible to receive gastro-protection to prevent the development of gastrointestinal ulcers, which was not reported in the ICSRs. Moreover, according to ibuprofen SmPC, ibuprofen should have been avoided in combination with low-dose acetylsalicylic acid, since this may increase the risk of adverse reactions due to its ability of inhibiting the effect of low-dose acetylsalicylic acid on platelet aggregation when they are administrated concomitantly.

Other clinical scenarios involving critical criteria related to patient behavior included four cases (4/19 critical criteria) for which it was reported “self-medication with non-over-the-counter,” of which three cases involving the use of non-therapeutic dosage of the medication and one case with inappropriate duration of the treatment if compared to the maximum duration reported in the SmPC. In two ICSRs (2/19 critical criteria) instead, it was reported the re-use of ketoprofen despite the case had medical history of hypersensitivity reaction to ketoprofen. Moreover, in three cases (3/19 critical criteria) the duration of the treatment with the over-the-counter medical product containing ibuprofen exceeded those suggested in the SmPC, of which 2 out of 3 cases with concurrent use of another NSAIDs that was listed in the SmPC as giving a potential drug-drug interaction. Additionally, in two ICSRs (2/19 critical criteria) it was reported a self-medication with a not therapeutic dosage of over-the-counter medical product containing ketoprofen. Finally, in two ICSRs, it was reported the sublingual administration of an oral formulation of ibuprofen 200 mg (1/19 critical criteria), and an infant accidentally ingest tablets of diclofenac 200 mg per os (1/19 critical criteria). All preventable cases with patient behavior critical criteria are shown in Supplementary Table 2.

### Sensitivity analysis - preventable events involving NSAIDs that did not require medical prescription during the study period

In all, 17 out 94 (18.1%) preventable cases involved medical products sold as over-the-counter containing ibuprofen (12/17 cases), ketoprofen (4/17 cases) or naproxen (1/17 cases). Both critical criteria related to patient behavior (32/72 critical criteria), as those related to healthcare professionals' practices (42/72 critical criteria) were detected.

The most detected clinical scenarios included eight cases that reported the use of a NSAID sold as over-the-counter concurrently to NSAIDs that required medical prescriptions, for which, according to SmPC of the over-the-counter medication, exists a labeled drug-drug interaction that increased the risk of gastrointestinal ulcer. Moreover, the concurrent use of multiple NSAIDs made the cases eligible to receive gastro-protection, which was not reported in the ICSRs.

Additionally, other two preventable cases reported the concurrent use of over-the-counter medical products containing ibuprofen and low-dose acetylsalicylic acid. According to SmPCs of both medications, the concurrent use of ibuprofen and low-dose acetylsalicylic acid is contraindicated and made the cases eligible to receive gastro-protection that was not reported in the ICSRs. All preventable cases involving NSAIDs sold as over-the-counter are presented in Supplementary Table 2.

### Preventable ADRs

Ninety-four preventable cases reported 181 signs or symptoms. As expected, the most involved system organ class was gastrointestinal disorders (142/181; 78.4%) with melena (31/142; 21.8%), gastric ulcer (19/142; 13.4%), and abdominal pain (17/142; 12.0%) as the most reported preferred term (Table 2). The complete list of ADRs reported in the preventable cases, including seriousness and outcomes, was shown in Supplementary Table 2.

**Table 2:** **Adverse drug reactions recognized in preventable cases involved NSAIDs, categorized by system organ class and three most reported preferred terms**.

**SOC**	***N***
**Gastrointestinal disorders**	**142**
Melena	31
Gastric ulcer	19
Abdominal pain	17
**Skin and subcutaneous tissue disorders**	**14**
Erythema	4
Urticaria	4
Angioedema	2
**Respiratory, thoracic and mediastinal disorders**	**8**
Dyspnea	5
Nasal congestion	1
Epistaxis	1
**Blood and lymphatic system disorders**	**4**
Anaemia	3
Hemorrhage	1
**General disorders and administration site conditions**	**4**
Asthenia	3
Incorrect drug administration route	1
**Investigations**	**3**
Hypotension	1
Abnormal coagulation tests	1
International normalized ratio increased	1
**Nervous system disorders**	**2**
Paresthesia	1
Pre-syncope	1
**Injury, poisoning and procedural complications**	**1**
Poisoning	1
**Metabolism and nutrition disorders**	**1**
Fluid retention	1
**Psychiatric disorders**	**1**
Restlessness	1
**Eye disorders**	**1**
Blurred vision	1
Total	181

*The adverse events were reported in Campania spontaneous reporting system from July 2012 – October 2016*.

*N, number of sign or symptoms; SOC, System Organ Class*.

## Discussion

To our knowledge, this is the first study that assessed the magnitude of preventability of ADRs involving NSAIDs as suspected drugs among those reported though Campania Region (Italy) spontaneous reporting system from July 2012 to October 2016. Despite evidence are available on the association between NSAIDs usage and the development of ADRs, to date, scarce are the evidence on magnitude of their preventability (European Medicine Agency, 2013). Even less is the information available on the detailed medication scenarios where preventable ADRs occurred, especially in Italy. This lack in knowledge made our results of great novelty because we were able to provide “real-world evidence” on both the widespread of NSAIDs medication error-induced preventable ADRs in our Regional territory and detailed information on the clinical scenario where preventable ADRs occurred. In particular, ninety-four preventable cases were found, with the majority of preventable cases having an age between 31 (1st quartile) and 60 (3rd quartile) years old. Preventable cases were detected in all age groups, including infants, adolescents, and elder. This result was expected considering that previous studies have evidenced a widespread inappropriate use of NSAIDs involving both young and elder users in the Italian population (Motola et al., 2004; Scarpignato et al., 2015; Cardile et al., 2016; Montagnani et al., 2016). Moreover, this result is in line with the drug utilization data published by AIFA which evidenced a higher use of preventive measure for gastrolesivity among patients with an age greater or equal to 65 years old that were concurrently exposed to multiple gastrolesive drugs (The Medicines Utilisation Monitoring Centre., 2015). We believe that the great magnitude of preventable NSAIDs-induced ADRs, as evidenced by the high reporting rate, could be related to the widespread use of this drug class in our Regional territory. It should be highlighted that although a reduced use of NSAIDs was observed in the last years in Campania Region, still a considerable pool of patients was found exposed to NSAIDs. In fact, it resulted that from 2012 to 2015, the use of NSAIDs in Campania Region was 9.6 (in 2012), 10.2 (in 2013), 11.0 (in 2014), and 10.7 DDD/1000 ab die (in 2015) higher than Italian average (The Medicines Utilisation Monitoring Centre., 2015). According to previous studies on Campania Region spontaneous reporting system (Parretta et al., 2014; Sessa et al., 2015, 2016a,b,c,d; Mascolo et al., 2016, 2017; Rafaniello et al., 2016; Scavone et al., 2016), also for ICSRs reporting NSAIDs, the hospital physicians was the primary source of reports for both preventable and not preventable cases. Interestingly, more than 50% of preventable cases required a hospitalization or prolongation of hospitalization with more than 85% of cases requiring pharmacological and/or non-pharmacological treatments, drug switch or withdrawal to treat ADRs. These results were related, in our opinion, to the severity of ADRs due to inappropriate use of NSAIDs. In fact, in the majority of cases, the inappropriate use of NSAIDs resulted in the occurrence of severe gastrointestinal ulcerative disorders leading to perforation or blood loss that for their severity required the hospitalization of the patient. Should be highlighted, in fact, that these events were found potentially related to the co-administration of multiple NSAIDs or to the co-administration of NSAIDs and corticosteroids, and low-dose acetylsalicylic acid without concurrent use of gastro-protective medications, and/or to incorrect administration duration. It is well known, that concurrent use of aforementioned combinations, as well as incorrect NSAIDs administration duration, may result in enhanced risk of gastrointestinal ulcers, bleeding and/or perforation, which onset typically occur rapidly (Gabriel et al., 1991; Hansen et al., 1996). Moreover, it is well recognized the importance of administrated gastro-protective agents to reduce the risk of NSAIDs-related gastrointestinal adverse events (Gabriel et al., 1991; Hansen et al., 1996; Leontiadis et al., 2007; Targownik et al., 2008; Rostom et al., 2009; Scheiman and Hindley, 2010). Based on recognized clinical scenarios, in our opinion, it is necessary to promote initiatives to increase the awareness of healthcare professionals on the risk associated with inappropriate use of NSAIDs in our regional territory. Since the majority of preventable cases involved inappropriate use of medical products that required medical prescriptions or over-the-counter medical products, these educational activities should target especially physicians and pharmacists. In particular, based on the clinical scenario identified in the preventable cases, it will be realized face-to-face courses aimed to share in an anonymized form the major criticisms found by revaluating ICSRs. Moreover, the divulgation of major criticisms found by revaluating ICSRs will be performed through our newsletter that cover a network of the major stakeholders involved in Pharmacovigilance in our Regional territory. Additionally, personalized feedback will be realized and sent to the reporter of preventable cases describing the clinical case and the risk associated to the inappropriate use of the NSAIDs. In all these activities, it will be highlighted the importance of administrating gastro-protective medications. Moreover, the importance of adhering SmPC regarding the indications of use (i.e., ketorolac), administration duration (i.e., ketorolac, diclofenac) and contraindications (i.e., cross-hypersensitivity to multiple NSAIDs, severe liver disorders, etc.). Moreover, it will be emphasized the importance of physicians as advisors for the patients on the correct use of prescribed NSAIDs. Additionally, it will be highlighted to physicians the necessity of performing medication reconciliation and checking potential drug-drug interactions through appropriate software (e.g., Micromedex® prior to the prescription/administration of NSAIDs, especially for patients undergoing to multiple pharmacological treatments (Rose et al., 2017). In collaboration with pharmacists, instead, it will be organized divulgation campaigns targeting citizens to inform them about the risk of inappropriate use of NSAIDs. These campaigns will involve a trained cohort of pharmacists that is representative of pharmacies located in Campania Region. This cohort was previously involved in other research project targeting citizens (Parretta et al., 2014). By performing educational activities to physicians and pharmacists and by divulgating the results of this study, we believe it should be theoretically possible to provide useful information to prevent medication errors involving over-the-counter drugs, which were reported in the 18.1% of preventable cases. In Italy, all medical products, including over-the-counter medication, need to be dispensed by pharmacists. While medication errors connected to the inappropriate use of medical products that required medical prescriptions are detectable by physicians, those involving over-the-counter medical products are potentially un-detectable by physicians, unless the patient informs the physicians. In this aspect, the pharmacist could play a key role. Pharmacist, in fact, could provide appropriate advising to patients regarding the appropriate use of over-the-counter medications, as well as the risk connected with their use with concurrent pharmacological treatments. Moreover, the pharmacist could provide precious advice regarding the risk of hypersensitivity reaction in patients with medical history of hypersensitivity reaction to the NSAIDs. Additionally, to provide support to the healthcare operators/citizens regarding pharmacological therapies, Campania Pharmacovigilance Regional activated a free advisory service accessible through email/phone call denominated “the clinical pharmacologist answer.” These initiatives are in line with those promoted by both European Medicine Agency and World Health Organization. In fact, aforementioned Organizations have recently emphasized the needing of proactive initiatives to identify ADRs occurred due to a medication error in order to achieve an effective risk minimization. In fact, being medication errors “modifiable factors” for ADRs, it is possible to act (e.g., education approaches) directly on them in order to reduce ADRs' burden (European Medicine Agency, 2011, 2013).

## Conclusion

By applying the biomedical methodology of P-Method, this study found ninety-four preventable cases among those involving NSAIDs sent through Campania Region spontaneous reporting system. Since preventing ADRs is a major priority for regulatory agencies we believe that educational activities for promoting an appropriate drug use will pay off in far fewer preventable adverse drug events, far less harm done to patients by medications, and far less cost to the nation's economy. Therefore, a call for action is necessary for regulatory agencies also trough Regional Pharmacovigilance Centers in order to promote initiatives able to increase the awareness of healthcare professionals on the risk associated with inappropriate use of NSAIDs and, more generally on the risk associated with the drugs more frequently used inappropriately.

## Author contributions

Drafting the work and revising it for important intellectual content: MS, AM, SG, LS, CR, AC, GD, CS, DC. Substantial contributions to the acquisition, analysis, or interpretation of data for the work: MS, AM, SG, LS, CR, AC, GD, CS, DC. Final approval of the version to be published: MS, AM, SG, LS, CR, AC, GD, CS, DC. Agreement to be accountable for all aspects of the work in ensuring that questions related to the accuracy or integrity of any part of the work are appropriately investigated and resolved: MS, AM, SG, LS, CR, AC, GD, CS, DC. Developed the concept and designed the study: MS and AM. Wrote the paper: MS and AM.

### Conflict of interest statement

The authors declare that the research was conducted in the absence of any commercial or financial relationships that could be construed as a potential conflict of interest. The reviewer DT and handling Editor declared their shared affiliation, and the handling Editor states that the process nevertheless met the standards of a fair and objective review.

## Footnotes

^1^International Conference on Harmonization E2D. Post-Approval Safety Data Management: Definitions and Standards for Expedited Reporting. Available online at: https://www.ich.org/fileadmin/Public_Web_Site/ICH_Products/Guidelines/Efficacy/E2D/Step4/E2D_Guideline.pdf (Accessed Apr 4, 2016).
