# Supplementary material for: Campania Preventability Assessment Committee (Italy): A Focus on the Preventability of Non-steroidal Anti-inflammatory Drugs' Adverse Drug Reactions
Source: Front Pharmacol. 2017 May 26;8:305. doi: 10.3389/fphar.2017.00305 (PMC5445158; doi:10.3389/fphar.2017.00305)
Supplement: Supplementary file 1 [file Table1.DOCX]

Supplementary Material

Article Title

Campania Preventability Assessment Committee (Italy): A Focus on the Preventability of Non-Steroidal Anti-Inflammatory Drugs Adverse Drug Reactions

Maurizio Sessa^1^* & Liberata Sportiello^1^, Annamaria Mascolo^1^, Cristina Scavone^1^, Silvia Gallipoli^1^, Gabriella di Mauro^1^, Daniela Cimmaruta^1^, Concetta Rafaniello^1^ & Annalisa Capuano^1^

^1^Campania Pharmacovigilance and Pharmacoepidemiology Regional Centre, Section of Pharmacology “L. Donatelli”, Department of Experimental Medicine, University of Campania “L. Vanvitelli”, Naples, Italy

*** Correspondence:**Maurizio Sessa
maurizio.sessa@unicampania.it

Keywords: Preventability_1_; Spontaneous Reporting System_2_; Pharmacovigilance_3_; Medication Errors_4_; Italy_5_; Humans_6_; Drug safety_7_; Adverse Event_8_

**Supplementary Table 1.** Critical criteria defined in the P Method**.**

|  | **Yes** | **No** | **Unknown** | **Not Applicable** |
| --- | --- | --- | --- | --- |
| **Critical criteria related to healthcare professionals’ practices** |  |  |  |  |
| 1. Incorrect dose?­­ |  |  |  |  |
| 2. Incorrect drug administration route? |  |  |  |  |
| 3. Incorrect drug administration duration? |  |  |  |  |
| 4. Incorrect drug dosage formulation administered? |  |  |  |  |
| 5. Expired drug administered? |  |  |  |  |
| 6. Incorrect storage of drug? |  |  |  |  |
| 7. Drug administration error (timing, rate, frequency, technique, preparation, manipulation, mixing)? |  |  |  |  |
| 8. Wrong indication? |  |  |  |  |
| 9. Inappropriate prescription according to the characteristics of the patient (age, sex, pregnancy, other)? |  |  |  |  |
| 10. Inappropriate prescription for patient’s underlying medical condition (renal failure, hepatic failure, etc.) or underlying pathology? |  |  |  |  |
| 11. Documented hypersensitivity to administered drug or drug class? |  |  |  |  |
| 12. Labelled drug–drug interaction? |  |  |  |  |
| 13. Therapeutic duplication (prescription of two or more medicines with similar ingredients)? |  |  |  |  |
| 14. Necessary medication not given? |  |  |  |  |
| 15. Withdrawal syndrome (due to abrupt discontinuation of treatment)? |  |  |  |  |
| 16. Incorrect laboratory or clinical monitoring of medicine? |  |  |  |  |
| **Critical criteria related to product/drug quality** |  |  |  |  |
| 17. Poor-quality drug administered? |  |  |  |  |
| 18. Counterfeit drug administered? |  |  |  |  |
| **Critical criteria related to patient behaviour** |  |  |  |  |
| 19. Non-compliance? |  |  |  |  |
| 20. Self-medication with non-Over-the-counter drug? |  |  |  |  |
