# Supplementary material for: Campania Preventability Assessment Committee (Italy): A Focus on the Preventability of Non-steroidal Anti-inflammatory Drugs' Adverse Drug Reactions
Source: Front Pharmacol. 2017 May 26;8:305. doi: 10.3389/fphar.2017.00305 (PMC5445158; doi:10.3389/fphar.2017.00305)
Supplement: Supplementary file 2 [file Table2.DOCX]

Supplementary Material

Article Title

Campania Preventability Assessment Committee (Italy): A Focus on the Preventability of Non-Steroidal Anti-Inflammatory Drugs Adverse Drug Reactions

Maurizio Sessa^1^* & Liberata Sportiello^1^, Annamaria Mascolo^1^, Cristina Scavone^1^, Silvia Gallipoli^1^, Gabriella di Mauro^1^, Daniela Cimmaruta^1^, Concetta Rafaniello^1^ & Annalisa Capuano^1^

^1^Campania Pharmacovigilance and Pharmacoepidemiology Regional Centre, Section of Pharmacology “L. Donatelli”, Department of Experimental Medicine, University of Campania “L. Vanvitelli”, Naples, Italy

*** Correspondence:**Maurizio Sessa
maurizio.sessa@unicampania.it

Keywords: Preventability_1_; Spontaneous Reporting System_2_; Pharmacovigilance_3_; Medication Errors_4_; Italy_5_; Humans_6_; Drug safety_7_; Adverse Event_8_

**Supplementary Table 2.** Case series of preventable cases reporting NSAIDs as suspected drug recognized in Campania spontaneous reporting system from July 2012 – October 2016**.**

| **Case demographic characteristics  Seriousness - Outcome  Causality Assessment – Adverse Drug Reaction Mechanism** | **Cause of preventability** | **Case description** | **Adverse drug reaction/s** |
| --- | --- | --- | --- |
| **Case 1**  Female 72 years old, European Serious – hospitalization - improvement  Possible Dose-related | Non-compliance (incorrect dose) | A case arbitrarily used a non-therapeutic dosage of ketoprofen (400 mg) per os for three days. According to the SmPC, the maximum dosage suggested for ketoprofen for the indication reported in the ICSR was 75 mg/os/die, to be administrated after the main meal. In the SmPC, it was clearly stated to not exceed the dosage of 75 mg per day. Ketoprofen 25 mg in combination with sucralfate was sold as over-the-counter during the entire study period, therefore, it could not be excluded a possible self-medication with a non-therapeutic dosage of ketoprofen. | Gastric ulcer, melena |
| **Case 2**  Male 68 years old, European Serious – hospitalization - not available  Possible  Dose-related | Incorrect drug administration duration, necessary medication not given | A case was treated with diclofenac 75 mg intramuscular injections for myalgia for three days, while the recommended maximum administration duration is two days according to SmPC. The case had concurrent treatment with low-dose acetylsalicylic acid 300 mg that according to SmPCs of both medications made the case eligible to receive gastro-protection to prevent the development of gastrointestinal ulcers, which was not reported in the ICSR. | Gastric ulcer, erosive duodenitis |
| **Case 3**  Male 62 years old, European Serious – hospitalization – recovered  Possible  Dose-related | Incorrect drug administration duration, necessary medication not given | A case was treated with diclofenac 50 mg for myalgia per os for six days, while the recommended maximum administration duration is two days according to SmPC. The case had concurrent treatment with low**-**dose acetylsalicylic acid 100 mg that according to SmPCs of both medications made the case eligible to receive gastro-protection to prevent the development of gastrointestinal ulcers, which was not reported in the ICSR. | Gastric ulcer, melena |
| **Case 4**  Male 43 years old, European Serious – hospitalization - improvement  Possible  Dose-related | Incorrect drug administration duration, wrong indication, labelled drug-drug interaction: ketorolac and diclofenac, therapeutic duplication, necessary medication not given | A case used for four days ketorolac 30 mg intramuscular injections and diclofenac 75 mg intramuscular injections for a backache. According to SmPCs of both medications, the recommended maximum administration duration for intramuscular formulations of ketorolac and diclofenac is two days. According to ketorolac SmPC, backache was not listed among recommended indications. According to SmPCs of both medications, concurrent use of multiple NSAIDs was listed as labelled drug-drug interaction that may result in enhanced risk of gastrointestinal adverse gastrointestinal effects (peptic ulcers, gastrointestinal bleeding and/or perforation). According to SmPC of both medications, patients with a high risk of gastrointestinal bleeding were eligible for receiving an appropriate gastro-protection to prevent the development of gastrointestinal ulcers, which the case did not receive. | Erosive gastritis, hematemesis |
| **Case 5**  Female 67 years old, European Serious – hospitalization - improvement  Possible  Dose-related | Necessary medication not given | A case was treated with diclofenac 100 mg per os for seven days for a backache. The case had concurrent treatment with low**-**dose acetylsalicylic acid 300 mg that According to SmPCs of both medications made the case eligible to receive gastro-protection to prevent the development of gastrointestinal ulcers, which was not reported in the ICSR. | Gastric ulcer, melena |
| **Case 6**  Male 71 years old, European Serious – hospitalization - improvement  Possible  Dose-related | Necessary medication not given | A case was treated with meloxicam 7.5 mg per os for arthralgia for eight days. The case had concurrent treatment with low**-**dose acetylsalicylic acid 100 mg that According to SmPCs of both medications made the case eligible to receive gastro-protection to prevent the development of gastrointestinal ulcers, which was not reported in the ICSR. | Duodenal ulcer, melena |
| **Case 7**  Female 62 years old, European Serious – hospitalization - not available  Possible  Dose-related | Necessary medication not given | A case was treated with piroxicam 20 mg intramuscular injections for arthralgia for four days. The case had concurrent treatment with low**-**dose acetylsalicylic acid 160 mg that According to SmPCs of both medications made the case eligible to receive gastro-protection to prevent the development of gastrointestinal ulcers, which was not reported in the ICSR. | Gastric ulcer, hematemesis, melena,  erosive duodenitis |
| **Case 8**  Male 51 years old, European Serious – hospitalization – improvement  Possible  Dose-related | Incorrect drug administration duration, wrong indication, labelled drug-drug interaction: ketorolac and diclofenac, therapeutic duplication, necessary medication not given | A case was treated with ketorolac 30 mg intramuscular injections and diclofenac 75 mg intramuscular injections for five days for low back pain. According to SmPCs of both medications, the recommended maximum administration duration is two days. According to SmPC, low back pain do not represent an approved indication of use for ketorolac. According to SmPCs of both medications, concurrent use of multiple NSAIDs was listed as labelled drug-drug interaction that may result in enhanced risk of gastrointestinal adverse gastrointestinal effects (peptic ulcers, gastrointestinal bleeding and/or perforation). According to SmPCs of both medications, concurrent use of multiple NSAIDs made the case eligible to receive gastro-protection to prevent the development of gastrointestinal ulcers, which was not reported in the ICSR. | Gastric ulcer, melena |
| **Case 9**  Female 44 years old, European Serious – life threatening - recovered  Probable  Susceptibility | Documented hypersensivity to administered drug or drug class | A case with a medical history of hypersensitivity reactions to un-specified NSAIDs was treated with ketoprofen 80 mg per os for a headache. The case developed angioedema and urticaria. According to SmPC, ketoprofen is contraindicated in patients who have a history of hypersensitivity reactions such as bronchospasm, asthmatic attacks, rhinitis, angioedema, urticaria or other allergic-type reactions to ketoprofen, any other ingredients in this medicine, or other NSAIDs. | Angioedema, urticaria |
| **Case 10**  Female 80 years old, European Serious – hospitalization - improvement  Possible  Dose-related | Non-compliance (necessary medication not given) | A case was exposed to ibuprofen 200 mg per os for two days. The day after the suspension of ibuprofen treatment, the case received betamethasone 0.5 mg per os. Ibuprofen 200 mg was dispensed as over-the-counter medication during the study period therefore, it could not be excluded a possible self-medication with ibuprofen. The case had a medical history of gastroresection due to a gastrointestinal ulcer. According to SmPCs of both medications, patients with a medical history of gastrointestinal ulcer were eligible to receive gastro-protection to prevent the development of gastrointestinal ulcers, which was not reported in the ICSR. | Melena, hemorrhagic stomatitis |
| **Case 11**  Female 37 years old, European  Not serious - recovered  Probable  Dose-related | Incorrect drug administration duration | A case was treated for eight days with an injectable formulation of ketoprofen 160 mg for low back pain. According to SmPC, the injectable formulation of ketoprofen 160 mg can be managed only by healthcare operators in a hospital setting and should be administered only for three days. | Abdominal pain |
| **Case 12**  Male 39 years old, European Not serious - improvement  Probable  Susceptibility | Documented hypersensivity to administered drug or drug class | A case with a medical history of hypersensitivity reactions to un-specified NSAIDs was treated with ketoprofen 160 mg for arthralgia. The case experienced lips’ oedema. According to SmPC, ketoprofen is contraindicated in patients who have a history of hypersensitivity reactions such as bronchospasm, asthmatic attacks, rhinitis, angioedema, urticaria or other allergic-type reactions to ketoprofen, any other ingredients in this medicine, or other NSAIDs. | Lips’ edema |
| **Case 13**  Male 74 years old, European  Serious – hospitalization - improvement  Possible  Dose-related | Incorrect drug administration duration, necessary medication not given | A case was treated with diclofenac 75 mg intramuscular injections for low back pain for three days, while the recommended maximum administration duration is two days according to SmPC. The case had concurrent treatment with low-dose acetylsalicylic acid 160 mg per os that According to SmPCs of both medications made the case eligible to receive gastro-protection to prevent the development of gastrointestinal ulcers, which was not reported in the ICSR. | Duodenal ulcer,  hematemesis |
| **Case 14**  Male 67 years old, European  Serious – hospitalization - improvement  Possible  Dose-related | Necessary medication not given | A case was treated with diclofenac 75 mg per os for arthralgia for three days. The case had concurrent treatment with low-dose acetylsalicylic acid 300 mg that According to SmPCs of both medications made the case eligible to receive gastro-protection to prevent the development of gastrointestinal ulcers, which was not reported in the ICSR. | Duodenal ulcer,  melena |
| **Case 15**  Female 26 years old, European  Not serious - recovered  Possible  Dose-related | Incorrect drug administration duration, labelled drug-drug interaction: betamethasone and diclofenac, necessary medication not given | A case was exposed to betamethasone intramuscular injections and diclofenac intramuscular injections for three days to treat low back pain, while in the SmPC, the recommended maximum administration duration of diclofenac intramuscular injections is two days. According to SmPCs of both medications, concurrent use NSAIDs with corticosteroids was listed as labelled drug-drug interaction that may result in enhanced risk of gastrointestinal adverse gastrointestinal effects (peptic ulcers, gastrointestinal bleeding and/or perforation). According to SmPCs of both medications, concurrent use of NSAIDs with corticosteroids made the case eligible to receive gastro-protection to prevent the development of gastrointestinal ulcers, which was not reported in the ICSR. | Abdominal pain |
| **Case 16**  Male 65 years old, European  Serious - other clinically relevant condition - not available  Possible  Dose-related | Wrong indication, necessary medication not given | A case was treated with diclofenac intramuscular injections for low back pain for one day. After discontinuing diclofenac, the patient received ketorolac intramuscular injections for low back pain for one day. For both treatments, the case had concurrent treatment with clopidogrel + low-dose acetylsalicylic acid that According to SmPCs of both medications made the case eligible to receive gastro-protection to prevent the development of gastrointestinal ulcers, which was not reported in the ICSR. The indication of use of ketorolac was low back pain, which represents a not recommended indication according to SmPC. | Duodenal ulcer |
| **Case 17**  Female 53 years old, European  Not serious - complete resolution  Possible  Dose-related | Incorrect drug administration duration | A case was co-exposed to the combination paracetamol + codeine per os and diclofenac intramuscular injections for eight days to treat low back pain. According to SmPC, the recommended maximum administration duration of the combination paracetamol + codeine per os is three days. | Abdominal pain |
| **Case 18**  Male 78 years old, European  Serious – other clinically relevant condition - complete resolution  Possible  Dose-related | Wrong indication, inappropriate prescription for patient’s underlying medical condition or underlying pathology | A case with liver cirrhosis was treated with an injectable formulation of ketorolac to treat arthrosis, which represents a not recommended indication according to SmPC. According to ketorolac SmPC, severe liver disorder represents a contraindication for the administration of ketorolac. | Gastrointestinal bleeding, hemorrhagic anaemia |
| **Case 19**  Female 72 years old, European  Serious – hospitalization - improvement  Possible  Dose-related | Necessary medication not given | A case with the concurrent use of low**-**dose acetylsalicylic acid 100 mg per os and piroxicam 20 mg intramuscular injections for six days. According to SmPCs of both medications, concurrent use of NSAIDs with low**-**dose acetylsalicylic acid made the case eligible to receive gastro-protection to prevent the development of gastrointestinal ulcers, which was not reported in the ICSR. | Abdominal pain, gastric ulcer, gastrointestinal bleeding, hematemesis, melena |
| **Case 20**  Female 53 years old, European  Serious – hospitalization - not available  Possible  Dose-related | Incorrect drug administration duration, wrong indication, labelled drug-drug interaction: ibuprofen and ketorolac, necessary medication not given | A case with the concurrent use of ibuprofen 800 mg per os for a headache and ketorolac 30 mg intramuscular injections for myalgia for six days. According to SmPC, the recommended maximum administration duration of ketorolac intramuscular injections is two days. The indication of use of ketorolac was myalgia, which represents a not recommended indication according to ketorolac SmPC. According to SmPCs of both medications, concurrent use of multiple NSAIDs was listed as labelled drug-drug interaction that may result in enhanced risk of gastrointestinal adverse gastrointestinal effects (peptic ulcers, gastrointestinal bleeding and/or perforation). | Gastric Ulcer |
| **Case 21**  Female 46 years old, European  Not serious - resolution with sequelae  Probable  Dose-related | Incorrect dose | A case used a non-therapeutic dosage of ketoprofen (340 mg/os/die for two days). In adults, according to SmPC, the maximum suggested dosage is 80 mg/os/ter in die, after the main meal. | Abdominal Pain |
| **Case 22**  Female 22 years old, European  Serious – hospitalization - improvement  Probable  Susceptibility | Documented hypersensivity to administered drug or drug class | A case with a medical history of hypersensitivity reaction to an un-specified NSAID was treated with ketoprofen 80 mg per os for pharyngitis. The case developed bronchoconstriction, dyspnea, lips’ edema, itch, and urticaria. According to SmPC, ketoprofen is contraindicated in patients who have a history of hypersensitivity reactions such as bronchospasm, asthmatic attacks, rhinitis, angioedema, urticaria or other allergic-type reactions to ketoprofen, any other ingredients in this medicine, or other NSAIDs. | Bronchoconstriction, dyspnea, lips’ edema, itch, urticaria |
| **Case 23**  Female 17 years old, European  Not serious - resolution with sequelae  Probable  Susceptibility | Wrong indication | A case used ketorolac 60 mg intramuscular injections for two days for a headache, which represent a not recommended indication according to SmPC. | Abdominal Pain, lips’ edema |
| **Case 24**  Male 59 years old, European  Serious – hospitalization - improvement  Possible  Dose-related | Incorrect drug administration duration , wrong indication, labelled drug-drug interaction: diclofenac and ketorolac, therapeutic duplication, necessary medication not given | A case with the concurrent use of diclofenac 75 mg intramuscular injections and ketorolac 30 mg intramuscular injections for arthralgia for nine days. According to SmPC, the recommended maximum administration duration of both medications is two days. The indication of use of ketorolac was myalgia, which represents a not recommended indication according to SmPC. According to SmPCs of both medications, concurrent use of multiple NSAIDs was listed as labelled drug-drug interaction that may result in enhanced risk of gastrointestinal adverse gastrointestinal effects (peptic ulcers, gastrointestinal bleeding and/or perforation). According to SmPCs of both medications, the concurrent use of multiple NSAIDs made the case eligible to receive gastro-protection to prevent the development of gastrointestinal ulcers, which was not reported in the ICSR. | Duodenal ulcer,  melena |
| **Case 25**  Male 19 years old, European  Not defined - improvement  Probable  Susceptibility | Documented hypersensivity to administered drug or drug class | A case with a medical history of hypersensitivity reaction to ibuprofen was treated with ibuprofen 600 mg per os. | Eritema, urticaria, lips’ edema, Itch |
| **Case 26**  Male 72 years old, European  Serious – hospitalization - improvement  Possible  Dose-related | Incorrect drug administration duration , wrong indication, labelled drug-drug interaction: ketorolac and diclofenac, therapeutic duplication, necessary medication not given | A case with the concurrent use of ketorolac 30 mg intramuscular injections and diclofenac 75 mg intramuscular injections for neck pain for six days. The recommended maximum administration duration of ketorolac and diclofenac intramuscular injections is two days According to SmPCs of both medications. The indication of use of ketorolac was neck pain, which represents a not recommended indication according to SmPC. According to SmPCs of both medications, concurrent use of multiple NSAIDs was listed as labelled drug-drug interaction that may result in enhanced risk of gastrointestinal adverse gastrointestinal effects (peptic ulcers, gastrointestinal bleeding and/or perforation). According to SmPCs of both medications, the concurrent use of multiple NSAIDs made the case eligible to receive gastro-protection to prevent the development of gastrointestinal ulcers, which was not reported in the ICSR. | Gastric ulcer,  hematemesis |
| **Case 27**  Male 62 years old, European  Serious – hospitalization - improvement  Possible  Dose-related | Necessary medication not given | A case received meloxicam 7.5 mg per os for knee pain for seven days. The case was in concurrent treatment with low**-**dose acetylsalicylic acid 300 mg per os. According to SmPCs of both medications, the concurrent use of NSAIDs and low- dose acetylsalicylic acid made the case eligible to receive gastro-protection to prevent the development of gastrointestinal ulcers, which was not reported in the ICSR. | Duodenal ulcer,  melena |
| **Case 28**  Male 52 years old, European  Serious – hospitalization - not available  Possible  Dose-related | Necessary medication not given | A case with the concurrent use of low**-**dose acetylsalicylic acid 100 mg per os for and ketoprofen 160 mg per os. According to SmPCs of both medications, the concurrent use of NSAIDs and low- dose acetylsalicylic acid made the case eligible to receive gastro-protection to prevent the development of gastrointestinal ulcers, which was not reported in the ICSR. | Rectorrhagia |
| **Case 29**  Female 37 years old, European  Serious – other clinically relevant condition - improvement  Possible  Dose-related | Incorrect drug administration duration, wrong indication, labelled drug-drug interaction: betamethasone and ketorolac, necessary medication not given | A case with the concurrent use of betamethasone intramuscular injections and ketorolac intramuscular injections for low back pain for fifteen days. According to SmPC of ketorolac, the recommended maximum administration duration for ketorolac is two days. The indication of use of ketorolac was low back pain, which represents a not recommended indication according to SmPC. According to SmPCs of both medications, concurrent use corticosteroids and NSAIDs was listed as labelled drug-drug interaction that may result in enhanced risk of gastrointestinal adverse gastrointestinal effects (peptic ulcers, gastrointestinal bleeding and/or perforation). According to SmPCs of both medications, the concurrent use of corticosteroids and NSAIDs made the case eligible to receive gastro-protection to prevent the development of gastrointestinal ulcers, which was not reported in the ICSR. | Abdominal pain, erosive gastritis, erosive duodenitis |
| **Case 30**  Male 52 years old, European  Serious – hospitalization - improvement  Possible  Dose-related | Incorrect drug administration duration | A case used diclofenac 150 mg intramuscular injections for a backache for five days, while the recommended maximum administration duration is two days according to SmPC of diclofenac intramuscular injections. | Duodenal ulcer, hematemesis, melena |
| **Case 31**  Male 62 years old, European  Serious – hospitalization - improvement  Possible  Dose-related | Non-compliance (labelled drug-drug interaction: acetylsalicylic acid and ibuprofen, necessary medication not given) | A Case with concurrent use of low**-**dose acetylsalicylic acid 300 mg per os and ibuprofen 800 mg per os for three days.  Ibuprofen administration is contraindicated with low**-**dose acetylsalicylic acid. According to SmPCs of both medications, concurrent use ibuprofen and low**-**dose acetylsalicylic was listed as labelled drug-drug interaction that may result in enhanced risk of gastrointestinal adverse gastrointestinal effects (peptic ulcers, gastrointestinal bleeding and/or perforation). According to SmPCs of both medications, the concurrent use of ibuprofen and low**-**dose acetylsalicylic made the case eligible to receive gastro-protection to prevent the development of gastrointestinal ulcers, which was not reported in the ICSR. | Gastric ulcer, hematemesis, melena |
| **Case 32**  Male 37 years old, European  Not serious - improvement  Probable  Susceptibility | Documented hypersensivity to administered drug or drug class | A case with a medical history of hypersensitivity reaction to ibuprofen was treated with ibuprofen 80 mg per os. | Dyspnea, hyperemia, nasal congestion, hyperemia of conjunctives |
| **Case 33**  Female 70 years old, European  Not serious – not available  Possible  Dose-related | Labelled drug-drug interaction: acetylsalicylic acid and ibuprofen, necessary medication not given | A Case with concurrent use of low**-**dose acetylsalicylic acid per os and ibuprofen per os for one month. Ibuprofen administration is contraindicated with low**-**dose acetylsalicylic acid. According to SmPCs of both medications, concurrent use ibuprofen and low**-**dose acetylsalicylic was listed as labelled drug-drug interaction that may result in enhanced risk of gastrointestinal adverse gastrointestinal effects (peptic ulcers, gastrointestinal bleeding and/or perforation). According to SmPCs of both medications, the concurrent use of ibuprofen and low**-**dose acetylsalicylic made the case eligible to receive gastro-protection to prevent the development of gastrointestinal ulcers, which was not reported in the ICSR. | Erosive gastritis |
| **Case 34**  Male 18 years old, European  Serious - other clinically relevant condition - not available  Possible  Dose-related | Wrong indication, labelled drug-drug interaction: ibuprofen and morniflumate, therapeutic duplication, necessary medication not given | A Case with concurrent use of ibuprofen per os, morniflumate per os and formoterol + budesonide aerosol to treat flu. According to SmPC, the flu is not an approved indication of use of formoterol + budesonide aerosol. According to SmPCs of both medications, concurrent use ibuprofen and morniflumate were listed as labelled drug-drug interaction that may result in enhanced risk of gastrointestinal adverse gastrointestinal effects (peptic ulcers, gastrointestinal bleeding and/or perforation). According to SmPCs of both medications, the concurrent use multiple NSAIDs made the case eligible to receive gastro-protection to prevent the development of gastrointestinal ulcers, which was not reported in the ICSR. | Erosive gastritis |
| **Case 35**  Female 53 years old, European  Serious – hospitalization - improvement  Possible  Dose-related | Incorrect drug administration duration , wrong indication, labelled drug-drug interaction: ketorolac and diclofenac, therapeutic duplication, necessary medication not given | A Case with concurrent use of ketorolac 75 mg intramuscular injections and diclofenac 75 mg intramuscular injections for five days to treat a backache. According to SmPC of both medications, the recommended maximum administration duration is two days. According to SmPC, backache was not an approved indication of use for ketorolac. According to SmPCs of both medications, concurrent use of multiple NSAIDs was listed as labelled drug-drug interaction that may result in enhanced risk of gastrointestinal adverse gastrointestinal effects (peptic ulcers, gastrointestinal bleeding and/or perforation). According to SmPCs of both medications, the concurrent use multiple NSAIDs made the case eligible to receive gastro-protection to prevent the development of gastrointestinal ulcers, which was not reported in the ICSR. | Gastric ulcer, melena |
| **Case 36**  Female 83 years old, European  Serious – hospitalization - improvement  Possible  Dose-related | Labelled drug-drug interaction: warfarin, diclofenac and ibuprofen, therapeutic duplication, necessary medication not given | A Case with concurrent use for three months of diclofenac per os and ibuprofen per os to treat pain in the shoulder. The case was treated with warfarin per os for permanent atrial fibrillation. According to SmPCs of both medications, concurrent use of warfarin and multiple NSAIDs was listed as labelled drug-drug interaction that may result in enhanced risk of bleeding. According to SmPCs of both medications, concurrent use of multiple NSAIDs was listed as labelled drug-drug interaction that may result in enhanced risk of gastrointestinal adverse gastrointestinal effects (peptic ulcers, gastrointestinal bleeding and/or perforation). According to SmPCs of both medications, the concurrent use multiple NSAIDs made the case eligible to receive gastro-protection to prevent the development of gastrointestinal ulcers, which was not reported in the ICSR. | Anaemia, asthenia, dyspnea, gastrointestinal hemorrhage, international normalized ratio increased, melena |
| **Case 37**  Male 63 years old, European  Serious – hospitalization - improvement  Possible  Dose-related | Labelled drug-drug interaction: nimesulide and diclofenac, therapeutic duplication, necessary medication not given | A case with the concurrent use of diclofenac 100 mg per os and nimesulide 100 mg per os for arthralgia for two days. According to SmPCs of both medications, concurrent use of multiple NSAIDs was listed as labelled drug-drug interaction that may result in enhanced risk of gastrointestinal adverse gastrointestinal effects (peptic ulcers, gastrointestinal bleeding and/or perforation). According to SmPCs of both medications, the concurrent use multiple NSAIDs made the case eligible to receive gastro-protection to prevent the development of gastrointestinal ulcers, which was not reported in the ICSR. | Duodenal ulcer, melena, hematemesis |
| **Case 38**  Female 26 years old, European  Not serious - improvement  Possible  Dose-related | Non-compliance, self-medication with non-over-the-counter drug | A case arbitrarily abused of nimesulide per os and ibuprofen for more than a month. Ibuprofen was sold as an over-the-counter drug during the study period. | Abdominal pain, hematemesis, melena |
| **Case 39**  Male 61 years old, European  Serious - other clinically relevant condition - improvement  Possible  Dose-related | Labelled drug-drug interaction: warfarin and ketoprofen | A case with the concurrent use of warfarin and ketoprofen. According to SmPCs of both medications, concurrent use of warfarin and ketoprofen was listed as labelled drug-drug interaction that may result in enhanced risk of bleeding. | Asthenia, abnormal coagulation tests |
| **Case 40**  Female 53 years old, European  Not serious – complete resolution  Probable  Dose-related | Wrong indication | A case used ketorolac 30 mg intramuscular injections as needed for abdominal colic. According to SmPC of ketorolac, abdominal colic was not a suggested indication. | Abdominal Pain |
| **Case 41**  Female 89 years old, European  Not serious – recovered  Possible  Dose-related | Labelled drug-drug interaction: warfarin and ketoprofen | A case with the concurrent use of warfarin and ketoprofen. According to SmPCs of both medications, concurrent use of warfarin and ketoprofen was listed as labelled drug-drug interaction that may result in enhanced risk of bleeding. | Epistaxis |
| **Case 42**  Male 42 years old, European  Serious - other clinically relevant condition - improvement  Probable  Dose-related | Incorrect drug administration duration, wrong indication | A case was treated for five days with an intravenous formulation of ketorolac for abdominal pain. According to SmPC, the intravenous formulation can be managed only by healthcare operators in a hospital setting and should be administered only for two days. According to SmPC, abdominal pain does not represent an approved indication of use for ketorolac. | Gastritis |
| **Case 43**  Male 50 years old, European  Serious – hospitalization - improvement  Probable  Dose-related | Incorrect drug administration duration | A case was treated with diclofenac 150 mg intramuscular injections for four days for neck pain. According to SmPC, the recommended maximum administration duration is two days. | Duodenal ulcer, melena |
| **Case 44**  Male 65 years old, European  Serious – hospitalization - improvement  Possible  Susceptibility | Documented hypersensitivity to administered drug or drug class | A case with a medical history of hypersensitivity reactions to acetylsalicylic acid was treated with ketoprofen 160 mg per rectal use for prostatitis. According to SmPC, ketoprofen is contraindicated in patients with a history of hypersensitivity reactions such as bronchospasm, asthmatic attacks, rhinitis, angioedema, urticaria or other allergic-type reactions to ketoprofen, any other ingredients in this medicine, or other NSAIDs. | Lips’ Edema |
| **Case 45**  Female 2 month, European  Serious – life threatening – improvement  Possible  Dose-related | Non-compliance | An infant accidentally took diclofenac 200 mg per os. The case developed restlessness. | Restlessness |
| **Case 46**  Male 44 years old, European  Serious – hospitalization – recovered  Possible  Dose-related | Incorrect dose, incorrect drug administration duration, wrong indication, labelled drug-drug interaction: diclofenac, ketorolac, betamethasone and nimesulide, therapeutic duplication, necessary medication not given | A case with the concurrent use of diclofenac 75 mg intramuscular injections, thiocolchicoside 4 mg intramuscular injections, betamethasone 4 mg intramuscular injections, nimesulide 400 mg per os and ketorolac 30 mg intramuscular injections for inflammation. The concurrent treatment with multiple NSAIDs, according to SmPCs of aforementioned medications, made the case eligible to receive gastro-protection to prevent the development of gastrointestinal ulcers, which was not reported in the ICSR. According to the SmPC, the suggested maximum dosage for nimesulide for the indication reported in the ICSR was 200 mg/os/die, to be administrated after the main meal. The case used thiocolchicoside intramuscular formulations for twenty days. According to SmPC of thiocolchicoside, the recommended maximum administration duration for intramuscular formulations is five days. According to ketorolac SmPC, ketorolac should be used for renal colic, while the indication reported in the ICSR was not listed among recommended indications. According to SmPCs of aforementioned medications, concurrent use of multiple NSAIDs was listed as labelled drug-drug interaction that may result in enhanced risk of gastrointestinal adverse gastrointestinal effects (peptic ulcers, gastrointestinal bleeding and/or perforation). | Anaemia, erosive gastritis, melena, rectorrhagia, erosive duodenitis |
| **Case 47**  Female 79 years old, European  Serious – hospitalization - improvement  Possible  Dose-related | Non-compliance (necessary medication not given) | A case arbitrarily used ibuprofen 200 mg per os for four days for throat irritation. Ibuprofen 200 mg was dispensed as over-the-counter medication during the study period. The case had concurrent treatment with low-dose acetylsalicylic acid 100 mg that according to SmPCs of both medications made the case eligible to receive gastro-protection to prevent the development of gastrointestinal ulcers, which was not reported in the ICSR. | Erosive gastritis , hematemesis |
| **Case 48**  Male 53 years old, European  Serious – hospitalization - improvement  Possible  Dose-related | Labelled drug-drug interaction: diclofenac and ibuprofen, necessary medication not given, non-compliance | A case with the concurrent use of diclofenac 100 mg per os and ibuprofen 800 mg per os for four days. According to SmPCs of both medications, concurrent use of multiple NSAIDs was listed as labelled drug-drug interaction that may result in enhanced risk of gastrointestinal adverse gastrointestinal effects (peptic ulcers, gastrointestinal bleeding and/or perforation). According to SmPCs of both medications, concurrent use of multiple NSAIDs made the case eligible to receive gastro-protection to prevent the development of gastrointestinal ulcers, which was not reported in the ICSR. Ibuprofen 800 mg was dispensed as over-the-counter medication during the study period. | Duodenal ulcer |
| **Case 49**  Male 73 years old, European  Serious – hospitalization - improvement  Possible  Dose-related | Necessary medication not given | A case was treated with ketoprofen 160 mg per os for a headache for five days. The case had concurrent treatment with low-dose acetylsalicylic acid 100 mg that According to SmPCs of both medications made the case eligible to receive gastro-protection to prevent the development of gastrointestinal ulcers, which was not reported in the ICSR. | Duodenal ulcer, melena |
| **Case 50**  Male 59 years old, European  Serious – hospitalization – not available  Possible  Dose-related | Wrong indication, necessary medication not given | A case was treated with ketorolac 60 mg intramuscular injections for a backache. According to ketorolac SmPC, backache was not listed among recommended indications. The case had concurrent treatment with low-dose acetylsalicylic acid 100 mg that According to SmPCs of both medications made the case eligible to receive gastro-protection to prevent the development of gastrointestinal ulcers, which was not reported in the ICSR. | Gastric ulcer |
| **Case 51**  Female 51 years old, European  Serious – hospitalization – improvement  Possible  Dose-related | Incorrect drug administration duration, wrong indication | A case received ketorolac 30 mg intramuscular injections for neck pain for three days. According to ketorolac SmPC, the recommended maximum administration duration for intramuscular formulations is two days and neck pain was not listed among recommended indications. | Duodenal ulcer, hematemesis |
| **Case 52**  Male 64 years old, European  Serious – hospitalization – resolution with sequelae  Possible  Dose-related | Self-medication with non-over-the-counter drug | A case with self-medication with ketorolac. Ketorolac cannot be dispensed without medical prescription during the study period. | Gastric perforation |
| **Case 53**  Male 72 years old, European  Serious – hospitalization - improvement  Possible  Dose-related | Necessary medication not given | A case was treated with diclofenac 100 mg per os for two days. The case had concurrent treatment with low-dose acetylsalicylic acid 160 mg that According to SmPCs of both medications made the case eligible to receive gastro-protection to prevent the development of gastrointestinal ulcers, which was not reported in the ICSR. | Duodenal ulcer |
| **Case 54**  Female 67 years old, European  Serious – hospitalization - improvement  Possible  Dose-related | Incorrect drug administration duration, wrong indication, labelled drug-drug interaction: betamethasone and ketorolac, necessary medication not given | A case with the concurrent use of ketorolac 30 mg intramuscular injections, betamethasone 4 mg intramuscular injections and tizanidine 4 mg intramuscular injections for eleven days for scleroderma. According to ketorolac SmPCs, the recommended maximum administration duration for intramuscular formulations is two days and scleroderma was not listed among recommended indications. According to SmPCs of aforementioned medications, concurrent use of ketorolac and betamethasone was listed as labelled drug-drug interaction that may result in enhanced risk of gastrointestinal adverse gastrointestinal effects (peptic ulcers, gastrointestinal bleeding and/or perforation). According to SmPCs of ketorolac and betamethasone, concurrent use of NSAIDs and corticosteroids made the case eligible to receive gastro-protection to prevent the development of gastrointestinal ulcers, which was not reported in the ICSR. | Melena |
| **Case 55**  Female 51 years old, European  Serious – hospitalization - improvement  Possible  Dose-related | Incorrect drug administration duration, wrong indication, labelled drug-drug interaction: diclofenac and ketorolac, therapeutic duplication, necessary medication not given | A case was treated for four days with an intravenous formulation of ketorolac for myalgia and diclofenac 75 mg intramuscular injections for an unknown therapeutic indication. According to ketorolac SmPC, only healthcare operators in a hospital setting can manage the intravenous formulation and myalgia was not an approved indication of use for ketorolac. According to SmPCs of both medications, the recommended maximum administration duration for diclofenac intramuscular formulations and ketorolac intravenous formulation is two days. According to SmPCs of both medications, concurrent use of multiple NSAIDs was listed as labelled drug-drug interaction that may result in enhanced risk of gastrointestinal adverse gastrointestinal effects (peptic ulcers, gastrointestinal bleeding and/or perforation). According to SmPCs of both medications, concurrent use of multiple NSAIDs made the case eligible to receive gastro-protection to prevent the development of gastrointestinal ulcers, which was not reported in the ICSR. | Gastric ulcer |
| **Case 56**  Female 45 years old, European  Serious – hospitalization - improvement  Possible  Dose-related | Inappropriate prescription for patient’s underlying medical condition or underlying pathology | A case was treated with diclofenac 75 mg intramuscular injections. The case had a medical history of erosive gastroduodenitis. According to diclofenac SmPC, erosive gastroduodenitis represents a contraindication for the administration of diclofenac. | Abdominal pain |
| **Case 57**  Male 30 years old, European  Serious – hospitalization – not available  Possible  Dose-related | Incorrect drug administration duration, labelled drug-drug interaction: nimesulide and ketorolac, necessary medication not given | A case was treated for three days with an intravenous formulation of ketorolac, an intravenous formulation of ceftriaxone 75 mg and nimesulide 100 mg per os. According to ketorolac SmPC, only healthcare operators in a hospital setting can manage the intravenous formulation and the recommended maximum administration duration is two days. According to SmPCs of ketorolac and nimesulide, concurrent use of multiple NSAIDs was listed as labelled drug-drug interaction that may result in enhanced risk of gastrointestinal adverse gastrointestinal effects (peptic ulcers, gastrointestinal bleeding and/or perforation). According to SmPCs of both medications, concurrent use of multiple NSAIDs made the case eligible to receive gastro-protection to prevent the development of gastrointestinal ulcers, which was not reported in the ICSR. | Melena, rectorrhagia |
| **Case 58**  Male 74 years old, European  Serious – other clinically relevant condition – not available  Possible  Dose-related | Inappropriate prescription for patient’s underlying medical condition or underlying pathology | A case with liver cirrhosis was treated with an injectable formulation of diclofenac 150 mg per os. According to diclofenac SmPC, the severe liver disorder was a contraindication for the administration of diclofenac. | Anaemia, melena |
| **Case 59**  Female 44 years old, European  Serious – hospitalization – improvement  Possible  Dose-related | Labelled drug-drug interaction: nimesulide and diclofenac, therapeutic duplication, necessary medication not given | A case with the concurrent use of diclofenac 100 mg per os and nimesulide 200 mg per os for acute neck pain for two days. According to SmPCs of both medications, concurrent use of multiple NSAIDs was listed as labelled drug-drug interaction that may result in enhanced risk of gastrointestinal adverse gastrointestinal effects (peptic ulcers, gastrointestinal bleeding and/or perforation). According to SmPCs of both medications, concurrent use of multiple NSAIDs made the case eligible to receive gastro-protection to prevent the development of gastrointestinal ulcers, which was not reported in the ICSR. | Duodenal ulcer, melena |
| **Case 60**  Male 56 years old, European  Serious – hospitalization – improvement  Possible  Dose-related | Incorrect drug administration duration, wrong indication | A case received ketorolac intramuscular injections for low back pain for three days. According to ketorolac SmPC, the recommended maximum administration duration for intramuscular formulations is two days and low back pain was not listed among recommended indications. | Duodenitis, gastric ulcer, gastritis, melena |
| **Case 61**  Male 33 years old, European  Not serious – improvement  Probable  Dose-related | Self-medication with non-over-the-counter drug | A case arbitrarily used a non-therapeutic dosage of ketoprofen (1920 mg) per os. In adults, according to SmPC, the maximum suggested dosage is ketoprofen 240 mg/os/die, after the main meal. Ketoprofen was dispensed with a repeatable recipe during the study period. | Abdominal pain |
| **Case 62**  Male 75 years old, European  Serious – other clinically relevant condition – improvement  Possible  Dose-related | Necessary medication not given | A case was treated with diclofenac 75 mg per os. The case had concurrent treatment with low-dose acetylsalicylic acid 100 mg that according to SmPCs of both medications made the case eligible to receive gastro-protection to prevent the development of gastrointestinal ulcers, which was not reported in the ICSR. | Gastric disorder |
| **Case 63**  Female 37 years old, European  Serious – other clinically relevant condition – improvement  Possible  Dose-related | Incorrect dose, labelled drug-drug interaction: nimesulide and ketoprofen, necessary medication not given | A case with the concurrent use of nimesulide 300 mg per os and ketoprofen 80 mg per os. In adults, according to SmPC, the suggested maximum dosage for nimesulide is 200 mg/os/die, after the main meal. According to SmPCs of both medications, concurrent use of multiple NSAIDs was listed as labelled drug-drug interaction that may result in enhanced risk of gastrointestinal adverse gastrointestinal effects (peptic ulcers, gastrointestinal bleeding and/or perforation). According to SmPCs of both medications, concurrent use of multiple NSAIDs made the case eligible to receive gastro-protection to prevent the development of gastrointestinal ulcers, which was not reported in the ICSR. | Abdominal pain |
| **Case 64**  Male 66 years old, European  Serious – hospitalization – improvement  Possible  Dose-related | Incorrect drug administration duration, labelled drug-drug interaction: diclofenac and betamethasone, necessary medication not given | A case with the concurrent use of diclofenac 75 mg intramuscular injections, thiocolchicoside 4 mg intramuscular injections and betamethasone 4 mg intramuscular injections. The case used thiocolchicoside intramuscular injections for twenty-two days. According to SmPCs of thiocolchicoside, the recommended maximum administration duration for intramuscular formulations is five days. According to SmPCs of both medications, concurrent use of diclofenac and betamethasone was listed as labelled drug-drug interaction that may result in enhanced risk of gastrointestinal adverse gastrointestinal effects (peptic ulcers, gastrointestinal bleeding and/or perforation). According to SmPCs of both medications, concurrent use of diclofenac and betamethasone made the case eligible to receive gastro-protection to prevent the development of gastrointestinal ulcers, which was not reported in the ICSR. | Abdominal pain |
| **Case 65**  Male 46 years old, European  Not serious – improvement  Probable  Dose-related | Wrong indication | A case used ketorolac 30 mg intramuscular injections for pain due to fracture, which represents a not recommended indication according to SmPC. | Abdominal pain |
| **Case 66**  Male 65 years old, European  Serious – hospitalization – improvement  Possible  Dose-related | Necessary medication not given | A case was treated with diclofenac 150 mg per os for five days. The case had concurrent treatment with low-dose acetylsalicylic acid 100 mg that According to SmPCs of both medications made the case eligible to receive gastro-protection to prevent the development of gastrointestinal ulcers, which was not reported in the ICSR. | Gastric ulcer, melena |
| **Case 67**  Male 56 years old, European  Serious – other clinically relevant condition – improvement  Possible  Dose-related | Labelled drug-drug interaction: diclofenac and betamethasone, necessary medication not given | A case with the concurrent use of diclofenac 75 mg intramuscular injections, and betamethasone 4 mg intramuscular injections. According to SmPCs of both medications, concurrent use of diclofenac and betamethasone was listed as labelled drug-drug interaction that may result in enhanced risk of gastrointestinal adverse gastrointestinal effects (peptic ulcers, gastrointestinal bleeding and/or perforation). According to SmPCs of both medications, concurrent use of diclofenac and betamethasone made the case eligible to receive gastro-protection to prevent the development of gastrointestinal ulcers, which was not reported in the ICSR. | Gastric Ulcer |
| **Case 68**  Female 55 years old, European  Serious – hospitalization – not recovered yet  Possible  Dose-related | Non-compliance, self-medication with non-over-the-counter drug | A case with self-medication with a non-therapeutic dose of paracetamol, ketoprofen, acetylsalicylic acid and paroxetine. Ketoprofen and paroxetine required medical prescription during the study period. Paracetamol and acetylsalicylic acid were dispensed as over-the-counter medication during the study period. | Poisoning |
| **Case 69**  Female 17 years old, European  Serious – life threatening – resolution with sequelae  Probable  Dose-related | Incorrect dose, labelled drug-drug interaction: nimesulide and ketoprofen, therapeutic duplication, necessary medication not given | A case with the concurrent use of nimesulide 300 mg per os and ketoprofen 160 mg per os for a headache. In adults, according to nimesulide SmPC, the maximum suggested dosage is 200 mg/os/die, after the main meal. According to SmPCs of both medications, concurrent use of multiple NSAIDs was listed as labelled drug-drug interaction that may result in enhanced risk of gastrointestinal adverse gastrointestinal effects (peptic ulcers, gastrointestinal bleeding and/or perforation). According to SmPCs of both medications, concurrent use of multiple NSAIDs made the case eligible to receive gastro-protection to prevent the development of gastrointestinal ulcers, which was not reported in the ICSR. | Gastritis |
| **Case 70**  Female 50 years old, European  Undefined – not available  Probable  Unknown | Incorrect drug administration duration | A case was treated for three days with an injectable formulation of diclofenac 75 mg for musculoskeletal pain. According to diclofenac SmPC, the injectable formulation of diclofenac 75 mg should be administered only for two days. | Fluid retention |
| **Case 71**  Male 48 years old, European  Not serious –improvement  Possible  Dose-related | Labelled drug-drug interaction: diclofenac and betamethasone, necessary medication not given | A case with the concurrent use of diclofenac 150 mg intramuscular injections, and betamethasone 3 mg intramuscular injections for low back pain for two days. According to SmPCs of both medications, concurrent use of diclofenac and betamethasone was listed as labelled drug-drug interaction that may result in enhanced risk of gastrointestinal adverse gastrointestinal effects (peptic ulcers, gastrointestinal bleeding and/or perforation). According to SmPCs of both medications, concurrent use of diclofenac and betamethasone made the case eligible to receive gastro-protection to prevent the development of gastrointestinal ulcers, which was not reported in the ICSR. | Abdominal pain |
| **Case 72**  Female 49 years old, European  Serious – other clinically relevant condition – improvement  Possible  Dose-related | Incorrect dose, wrong indication, labelled drug-drug interaction: clopidogrel + acetylsalicylic acid and ketorolac, necessary medication not given | A case treated with ketorolac 10 mg intramuscular injections for pain. The case had concurrent treatment with low-dose acetylsalicylic acid 100 mg and clopidogrel + acetylsalicylic acid 175 mg, that according to SmPCs of both medications made the case eligible to receive gastro-protection to prevent the development of gastrointestinal ulcers, which was not reported in the ICSR. In adults, according to SmPC, the maximum suggested dosage is clopidogrel + acetylsalicylic acid is 75 mg. According to ketorolac SmPCs, the pain was not listed among recommended indications. According to SmPCs of both medications, concurrent use of clopidogrel + acetylsalicylic acid and ketorolac was listed as labelled drug-drug interaction that may result in enhanced risk of gastrointestinal bleeding. | Erosive gastritis |
| **Case 73**  Female 62 years old, European  Not serious – not available  Possible  Unknown | Non-compliance (incorrect drug administration duration) | A case used for over a month a transdermal formulation of ibuprofen 136 mg for musculoskeletal pain. According to SmPC, the transdermal formulation of ibuprofen 136 mg should be administered only for fourteen days. Transdermal formulation of ibuprofen 136 mg was dispensed as over-the-counter medication during the study period. | Dysgeusia, paresthesia |
| **Case 74**  Female 39 years old, European  Not serious – improvement  Probable  Susceptibility | Non-compliance (documented hypersensitivity to administered drug or drug class) | A case with the medical history of hypersensitivity reactions to ketoprofen was treated with ibuprofen 400 mg for a headache. According to SmPC, ibuprofen is contraindicated in patients who have a history of hypersensitivity reactions such as bronchospasm, asthmatic attacks, rhinitis, angioedema, urticaria or other allergic-type reactions to ibuprofen, any other ingredients in this medicine, or other NSAIDs. Ibuprofen 400 mg was dispensed as over-the-counter medication during the study period. | Erythema, itch |
| **Case 75**  Male 40 years old, European  Not serious – improvement  Probable  Unknown | Non-compliance (incorrect drug administration route) | A case used ibuprofen 200 mg per os, as sublingual formulation. Ibuprofen 200 mg was dispensed as over-the-counter medication during the study period. | Dyspepsia, stomatitis, Incorrect drug administration route |
| **Case 76**  Female 74 years old, European  Serious – hospitalization - improvement  Possible  Dose-related | Incorrect drug administration duration, labelled drug-drug interaction: diclofenac and ketorolac, necessary medication not given | A case used for seven days ketorolac 30 mg intramuscular injections for unknown indication and diclofenac 75 mg intramuscular injections for a backache. The case had concurrent treatment with multiple NSAIDs that according to SmPCs of both medications made the case eligible to receive gastro-protection to prevent the development of gastrointestinal ulcers, which was not reported in the ICSR. According to SmPCs of both medications, the recommended maximum administration duration of intramuscular formulations of ketorolac and diclofenac is two days. According to SmPCs of both medications, concurrent use of multiple NSAIDs was listed as labelled drug-drug interaction that may result in enhanced risk of gastrointestinal adverse gastrointestinal effects (peptic ulcers, gastrointestinal bleeding and/or perforation). | Duodenal ulcer, melena |
| **Case 77**  Female 55 years old, European  Serious – hospitalization – complete resolution  Probable  Susceptibility | Documented hypersensitivity to administered drug or drug class | A case with medical a history of hypersensitivity reactions to diclofenac was treated with diclofenac 150 mg for low back pain. According to SmPC, diclofenac is contraindicated in patients who have a history of hypersensitivity reactions such as bronchospasm, asthmatic attacks, rhinitis, angioedema, urticaria or other allergic-type reactions to diclofenac, any other ingredients in this medicine, or other NSAIDs. | Angioedema, dyspnea, erythema, urticaria |
| **Case 78**  Male 54 years old, European  Serious – hospitalization - improvement  Possible  Dose-related | Incorrect dose, necessary medication not given | A case used diclofenac 200 mg/Kg per os for myalgia for two days. The case had concurrent treatment with low-dose acetylsalicylic acid 160 mg/kg that According to SmPCs of both medications made the case eligible to receive gastro-protection to prevent the development of gastrointestinal ulcers, which was not reported in the ICSR. According to the SmPC, the suggested maximum dosage for diclofenac for the indication reported in the ICSR was 200 mg/os/die. According to the SmPC, the suggested maximum dosage for low-dose acetylsalicylic acid for the indication reported in the ICSR was 160 mg/os/die. | Gastric ulcer with hemorrhage, melena |
| **Case 79**  Female 24 years old, European  Serious – other clinically relevant condition – not available  Possible  Dose-related | Incorrect drug administration duration, wrong indication, labelled drug-drug interaction: ketoprofen and ketorolac, therapeutic duplication, necessary medication not given | A case was treated with ketorolac 10 mg intramuscular injections and ketoprofen 80 mg per os for three days for a headache. According to ketorolac SmPCs, the recommended maximum administration duration is two days and headache do not represent an approved indication of use. According to SmPCs of both medications, concurrent use of multiple NSAIDs was listed as labelled drug-drug interaction that may result in enhanced risk of gastrointestinal adverse gastrointestinal effects (peptic ulcers, gastrointestinal bleeding and/or perforation). According to SmPCs of both medications, concurrent use of multiple NSAIDs made the case eligible to receive gastro-protection to prevent the development of gastrointestinal ulcers, which was not reported in the ICSR. | Melena, rectorrhagia |
| **Case 80**  Male 76 years old, European  Serious – other clinically relevant condition – not available  Possible  Dose-related | Incorrect drug administration duration, wrong indication, necessary medication not given | A case was treated with ketorolac 20 drops per os for pain for six days. According to ketorolac SmPCs, the recommended maximum administration duration is five days and pain do not represent an approved indication of use. The case had concurrent treatment with low-dose acetylsalicylic acid 100 mg that according to SmPCs of both medications made the case eligible to receive gastro-protection to prevent the development of gastrointestinal ulcers, which was not reported in the ICSR. | Gastroduodenal ulcer |
| **Case 81**  Female 68 years old, European  Serious – other clinically relevant condition – not available  Possible  Dose-related | Incorrect drug administration duration, labelled drug-drug interaction: diclofenac and nimesulide, therapeutic duplication, necessary medication not given | A case was treated with diclofenac 75 mg intramuscular injections and nimesulide 200 mg per os for fourteen days for arthralgia. According to diclofenac SmPCs, the recommended maximum administration duration is two days. According to SmPCs of both medications, concurrent use of multiple NSAIDs was listed as labelled drug-drug interaction that may result in enhanced risk of gastrointestinal adverse gastrointestinal effects (peptic ulcers, gastrointestinal bleeding and/or perforation). The case had concurrent treatment with low-dose acetylsalicylic acid 100 mg that according to SmPCs of both medications made the case eligible to receive gastro-protection to prevent the development of gastrointestinal ulcers, which was not reported in the ICSR. | Erosive gastritis |
| **Case 82**  Male 59 years old, European  Not serious – not available  Possible  Dose-related | Labelled drug-drug interaction: naproxen and morniflumate, necessary medication not given | A case with the concurrent use of morniflumate 700 mg per os for bronchitis for sixteen days and naproxen 500 mg per os for osteoarthritis for over a month. According to SmPCs of both medications, concurrent use of multiple NSAIDs was listed as labelled drug-drug interaction that may result in enhanced risk of gastrointestinal adverse gastrointestinal effects (peptic ulcers, gastrointestinal bleeding and/or perforation). According to SmPCs of both medications, concurrent use of multiple NSAIDs made the case eligible to receive gastro-protection to prevent the development of gastrointestinal ulcers, which was not reported in the ICSR. | Gastritis, esophagitis |
| **Case 83**  Female 45 years old, European  Serious – hospitalization - improvement  Possible  Dose-related | Labelled drug-drug interaction: acetylsalicylic acid and ibuprofen, necessary medication not given, non-compliance | A case with concurrent use for three days of ibuprofen 400 mg per os for menstrual cramps and diclofenac 75 mg per os for a backache. The case had concurrent treatment with low-dose acetylsalicylic acid 100 mg that according to SmPCs of both medications made the case eligible to receive gastro-protection to prevent the development of gastrointestinal ulcers, which was not reported in the ICSR. Ibuprofen is contraindicated with low**-**dose acetylsalicylic acid and was sold as over-the-counter during the entire study period. According to SmPCs of both medications, concurrent use of multiple NSAIDs was listed as labelled drug-drug interaction that may result in enhanced risk of gastrointestinal adverse gastrointestinal effects (peptic ulcers, gastrointestinal bleeding and/or perforation). | Duodenal ulcer with hemorrhage, melena |
| **Case 84**  Female 43 years old, European  Not serious – improvement  Possible  Dose-related | Labelled drug-drug interaction: ketorolac and dexamethasone, necessary medication not given | A case was treated with an intravenous formulation of ketorolac and an intravenous formulation of dexamethasone 4 mg for post-operative pain. According to ketorolac SmPC, only healthcare operators in a hospital setting can manage the intravenous formulation. According to SmPCs of both medications, concurrent use of ketorolac and dexamethasone was listed as labelled drug-drug interaction that may result in enhanced risk of gastrointestinal adverse gastrointestinal effects (peptic ulcers, gastrointestinal bleeding and/or perforation). According to SmPCs of both medications, concurrent use of ketorolac and dexamethasone made the case eligible to receive gastro-protection to prevent the development of gastrointestinal ulcers, which was not reported in the ICSR. | Abdominal pain |
| **Case 85**  61 years old, European  Serious – other clinically relevant condition – improvement  Possible  Dose-related | Incorrect drug administration duration, wrong indication | A case was treated with an intravenous formulation of ketorolac for pain for over a month. According to ketorolac SmPC, the recommended maximum administration duration is two days and pain do not represent an approved indication of use. | Melena |
| **Case 86**  Female 49 years old, European  Not serious – recovered  Possible  Dose-related | Labelled drug-drug interaction: ketoprofen, ibuprofen and metamizole, therapeutic duplication, necessary medication not given, non-compliance | A case with the concurrent use of ketoprofen, ibuprofen, and metamizole for a toothache. According to SmPCs of ketoprofen, ibuprofen and metamizole, concurrent use of multiple NSAIDs was listed as labelled drug-drug interaction that may result in enhanced risk of gastrointestinal adverse gastrointestinal effects (peptic ulcers, gastrointestinal bleeding and/or perforation). According to SmPCs of aforementioned medications, concurrent use of multiple NSAIDs made the case eligible to receive gastro-protection to prevent the development of gastrointestinal ulcers, which was not reported in the ICSR. Ibuprofen was dispensed as over-the-counter medication during the study period. | Abdominal pain, emesis |
| **Case 87**  Male 35 years old, European  Serious – life threatening – improvement  Probable  Susceptibility | Non-compliance (documented hypersensivity to administered drug or drug class) | A case with a medical history of hypersensitivity reactions to ketoprofen, acetylsalicylic acid, and nimesulide was treated with ibuprofen for a headache. According to ibuprofen SmPC, ibuprofen is contraindicated in patients who have a history of hypersensitivity reactions such as bronchospasm, asthmatic attacks, rhinitis, angioedema, urticaria or other allergic-type reactions to ibuprofen, any other ingredients in this medicine, or other NSAIDs. Ibuprofen was dispensed as over-the-counter medication during the study period. | Dyspnea, face edema, itch |
| **Case 88**  Female 30 years old, European  Serious – hospitalization – recovered  Probable  Susceptibility | Documented hypersensivity to administered drug or drug class | A case with a medical history of hypersensitivity reactions to diclofenac was treated with ketorolac for arthralgia. According to ketorolac SmPC, ketorolac is contraindicated in patients who have a history of hypersensitivity reactions such as bronchospasm, asthmatic attacks, rhinitis, angioedema, urticaria or other allergic-type reactions to ketorolac, any other ingredients in this medicine, or other NSAIDs. | Hypotension, pre-syncope, itch, rash, blurred vision |
| **Case 89**  Male 53 years old, European  Serious – hospitalization - improvement  Possible  Dose-related | Labelled drug-drug interaction: nimesulide, acetylsalicylic acid, prednisone and naproxen, therapeutic duplication, necessary medication not given, non-compliance | A case with the concurrent use of acetylsalicylic acid, prednisone, and naproxen. According to SmPCs of aforementioned medications, concurrent use of acetylsalicylic acid, prednisone, and naproxen was listed as labelled drug-drug interaction that may result in enhanced risk of gastrointestinal adverse gastrointestinal effects (peptic ulcers, gastrointestinal bleeding and/or perforation). According to SmPCs of aforementioned medications, concurrent use of acetylsalicylic acid, prednisone, and naproxen made the case eligible to receive gastro-protection to prevent the development of gastrointestinal ulcers, which was not reported in the ICSR. Naproxen was dispensed as over-the-counter medication during the study period. | Hemorrhagic gastric ulcer, gastritis, erosive duodenitis, hyperemia of gastrointestinal mucosa |
| **Case 90**  Female 13 years old, European  Not serious – complete resolution  Probable  Susceptibility | Documented hypersensivity to administered drug or drug class | A case with a medical history of hypersensitivity reactions to paracetamol and acetylsalicylic acid was treated with ketoprofen for a sore throat. According to SmPC, ketoprofen is contraindicated in patients who have a history of hypersensitivity reactions such as bronchospasm, asthmatic attacks, rhinitis, angioedema, urticaria or other allergic-type reactions to ketoprofen, any other ingredients in this medicine, or other NSAIDs. | Lips’ edema and itch |
| **Case 91**  Female 42 years old, European  Serious – hospitalization - improvement  Possible  Susceptibility | Documented hypersensivity to administered drug or drug class | A case with a medical history of hypersensitivity reactions to ketoprofen was treated with ketoprofen for a sore throat and pantoprazole for prophylaxis of ulcer drug induced. The case experienced asthenia, erythema, face’s edema and tongue’s edema. According to SmPC, ketoprofen is contraindicated in patients who have a history of hypersensitivity reactions such as bronchospasm, asthmatic attacks, rhinitis, angioedema, urticaria or other allergic-type reactions to ketoprofen, any other ingredients in this medicine, or other NSAIDs. | Asthenia, erythema, face edema, lips edema |
| **Case 92**  Male 58 years old, European  Serious – hospitalization - improvement  Possible  Dose-related | Labelled drug-drug interaction: ketoprofen and diclofenac, therapeutic duplication, necessary medication not given | A case with the concurrent use of diclofenac 100 mg per os and ketoprofen per os for eight days to treat low back pain. According to SmPCs of both medications, concurrent use of ketoprofen and diclofenac was listed as labelled drug-drug interaction that may result in enhanced risk of gastrointestinal adverse gastrointestinal effects (peptic ulcers, gastrointestinal bleeding and/or perforation). According to SmPCs of both medications, concurrent use of ketoprofen and diclofenac made the case eligible to receive gastro-protection to prevent the development of gastrointestinal ulcers, which was not reported in the ICSR. | Gastric ulcer, erosive gastritis |
| **Case 93**  Female 63 years old, European  Serious – hospitalization - improvement  Possible  Dose-related | Incorrect drug administration duration, wrong indication, labelled drug-drug interaction: ketorolac and ketoprofen, therapeutic duplication, necessary medication not given | A case was treated with ketorolac per os and ketoprofen per os for six days for pain. According to ketorolac SmPCs, the recommended maximum administration duration is five days and pain do not represent an approved indication of use. According to SmPCs of both medications, concurrent use of multiple NSAIDs was listed as labelled drug-drug interaction that may result in enhanced risk of gastrointestinal adverse gastrointestinal effects (peptic ulcers, gastrointestinal bleeding and/or perforation). According to SmPCs of both medications, concurrent use of multiple NSAIDs made the case eligible to receive gastro-protection to prevent the development of gastrointestinal ulcers, which was not reported in the ICSR. | Erosive gastritis |
| **Case 94**  Male 57 years old, European  Serious – hospitalization - improvement  Probable  Dose-related | Incorrect drug administration duration, wrong indication | A case was treated for three days with an injectable formulation of ketorolac for pain. According to SmPC, the recommended maximum administration duration is two days and pain do not represent an approved indication of use. | Gastritis Erosive |
